# Quantitative Impact Analysis of Climate Change on Residents’ Health Conditions with Improving Eco-Efficiency in China: A Machine Learning Perspective

**DOI:** 10.3390/ijerph182312842

**Published:** 2021-12-06

**Authors:** Xianning Wang, Zhengang Ma, Jingrong Dong

**Affiliations:** 1 School of Economics and Management, Chongqing Normal University, No. 37, Middle Road of University Town, Shapingba District, Chongqing 401331, China; 20170002@cqnu.edu.cn; 2 Regional Economics Applications Laboratory (REAL), University of Illinois at Urbana-Champaign, Champaign, IL 61801, USA; 3 Big Data Marketing Research and Applications Center of Chongqing Normal University, Chongqing 401331, China; 4 College of Life Sciences, Chongqing Normal University, Chongqing 401331, China; mzgcqnu@126.com

**Keywords:** resident health conditions, climate change, eco-efficiency, empirical evidence, Sensitivity Evaluation with Support Vector Machines

## Abstract

Climate change affects public health, and improving eco-efficiency means reducing the various pollutants that are the result of economic activities. This study provided empirical evidence of the quantitative impact analysis of climate change on the health conditions of residents across China due to improvements that have been made to eco-efficiency. First, the indicators that were collected present adequate graphical trends and regional differences with a priori evidence about their relationships to each other; second, the present study applied Sensitivity Evaluation with Support Vector Machines (SE-SVM) to Chinese provincial panel data, taking the Visits to Hospitals, Outpatients with Emergency Treatment, and Number of Inpatients as proxy variables for the health conditions of the residents in each area and temperature, humidity, precipitation, and sunshine as the climate change variables, simultaneously incorporating the calculated eco-efficiency with six controlling indicators; third, we compared in-sample forecasting to acquire the optimal model in order to conduct elasticity analysis. The results showed that (1) temperature, humidity, precipitation, and sunshine performed well in forecasting the health conditions of the residents and that climate change was a good forecaster for resident health conditions; (2) from the national perspective, climate change had a positive relationship with Visits to Hospitals and Outpatients with Emergency Treatment but a negative relationship with the Number of Inpatients; (3) An increase in regional eco-efficiency of 1% increase the need for Visits to Hospitals and Outpatients with Emergency Treatment by 0.2242% and 0.2688%, respectively, but decreased the Number of Inpatients by 0.6272%; (4) increasing the regional eco-efficiency did not show any positive effects for any individual region because a variety of local activities, resource endowment, and the level of medical technology available in each region played different roles. The main findings of the present study are helpful for decision makers who are trying to optimize policy formulation and implementation measures in the cross-domains of economic, environmental, and public health.

## 1. Introduction

Decision makers are constantly weighing the relationships between climate change, public health, and economic development with environmental sustainability (Kan et al. 2012 [1]. The quantitative impact analysis of climate change on public health when considering the human initiatives to improve eco-efficiency in China is a hot and important interdisciplinary topic. It is crucial to acquire empirical evidence in order for scholars, entrepreneurs, and local government to formulate relevant policies and to optimize safeguard measures. However, it is a challenging task to quantitatively obtain more accurate calculation results on the basis of local original data. Resident health conditions (**RHC**) are affected by both climate change (represented by the proxy variables temperature, precipitation, humidity, and sunshine) and human economic activities and their corresponding environmental changes (represented by regional eco-efficiency **REE** [2] and consider the economic or resource cost and waste gas, wastewater, solid waste, etc., comprehensively). Therefore, utilizing original data in China, this paper aims to provide a quantitative impact analysis of climate change on the health conditions of the residents in each area of the country as the result of improving the country’s eco-efficiency.

The literature review shows that the relevant research that can be referred to is very rich but that research that is focused on these quantitative relationships from a spatial provincial level is rare. Previously, scholars have devoted time to investigating climate change and human health [1,3,4,5,6] and have maintained that climate change affects the health status of residents from the perspective of the ecosystem [7,8]; living environment [9,10]; agricultural production, trade [11], and food [12,13]; disease transmission [4,14,15]; and natural emergencies, among others. Meanwhile, due to limited data availability, multiple factors, and the uncertainty relationship, multiple related research studies have focused on the micro-level and theoretical analysis, and there is lack of a comprehensive quantitative analysis removing of the unconsensual diversified representative indicators [1]. For example, climate change is able to impact public health through a range of pathways [5]. Patz et al. summarized and stated that a number of prevalent human diseases are related to climate fluctuations, such as cardiovascular mortality and respiratory illnesses that are affected by heat waves [4]. McMichael et al. indicated that there is epidemiological evidence or trends and various health problems that are related to the impact of climate variations [6]. Meanwhile, scholars have mostly paid attention to thermal stress, contagious disease, or extreme weather and have limited attention to future food yields or hunger prevalence.

This literature review also informed us that it is necessary to incorporate the role of improving regional eco-efficiency when computing the quantitative impact analysis. Based on the previous literature, **REE** refers to economic activities such as air pollution [16,17,18], water pollution [19], heavy metal pollution [20,21,22], solid waste pollution [23], etc., which impose great risk to resident health, achieving a low environmental cost. **RHC** can greatly benefit from a sustainable and circular environment [24], as these environmental features would allow the target of optimizing eco-efficiency to be maintained at a consistent pace. Additionally, **RHC** can be used to judge and provide feedback regarding their subsequent, far-reaching social benefits as they pertain to **REE** [25]. In general, considering that the positive impact of increasing **REE** has been generally recognized [2], it is time to take **RHC** as one ruler to further appraise REE performance quantitatively by means of existing sample data [26].

To achieve the goal, this paper emphasized the following aspects:

Firstly, this paper selected and determined more recognized indicators for Climate Change, Resident Health Conditions, and Regional Eco-efficiency and then collected provincial panel data for the different regions across China. Specifically, we chose to analyze the Visits to Hospitals (**VTH**), Outpatients with Emergency Treatment (**OWT**), and Number of Inpatients (**NOI**) as proxy variables for **RHC**; adopted temperature, humidity, precipitation, and sunshine for the climate change variables and calculated the **REE**; and we then chose the six most important control variables to present regional characteristics, such as local activities, resource endowment, and the level of medical technology in each area.

Second, this paper preliminarily judged the change trends and the relationships between the variables and analyzed the regional characteristics and spatial heterogeneity. This paper made a relationship diagram; measured the correlation coefficient and judged the cooperative relationship between the variables; and illustrated the current situation through descriptive statistical analysis (which mainly included the spatial characteristics and trend analysis) of **RHC as well as** climate change and **REE** in China.

Third, this paper referred to the uncertainty of the relationship from the machine learning perspective and built the **SE-SVM** vector machines by tackling uncertain mapping with many factors and computation power levels [2,27]. This paper modified Least Square-Support Vector Regression (**LS-SVR**) to tackle the limited data availability, multiple factors, and the uncertain relationship.

Fourth, this paper applied the collected data to draw relevant empirical results, including two parts: (1) Forecasting the resident health conditions using the effects of climate change after the **REE** in China had been improved and (2) measuring how climate change impacts resident health conditions when the **REE** in China is improved.

Finally, a discussion and summary of the main conclusions are presented, and policy recommendations are made.

## 2. Materials and Methods

### 2.1. Data and Variables

The main indicators were selected through the following principles: The first principle was data availability; high-quality and easy-to-access indicators were prioritized. The second principle was the representativeness of the indicators, and priority was given to statistical data that could reflect the true results of the indicators. The third is the descriptive ability in multiple dimensions, wherein a comprehensive perspective could be used to conduct an analysis. The fourth was the regional differences, which are able to reflect the differences in different provinces or cities as much as possible.

Approximately 30 Chinese provinces and cities were chosen as samples. The period represented by the data covers 1998 to 2017. The main variables for the form of a single scalar called SBM (Slacks-Based Measure) to calculate the **REE** By DEA-SOLVER Pro 5.0 are listed in Table 1 [2,28], and their main descriptive statistics are included. The **REE** become much higher in the eastern areas than they do in the western areas, and in 2017, the whole value level is 0.51. Additionally, the **REE** is shown to have improved stably from 1998 to 2017. Figure 2 provides its ranking and a comparison of 30 provinces and cities in China from 1998–2017.

**Figure 1:**
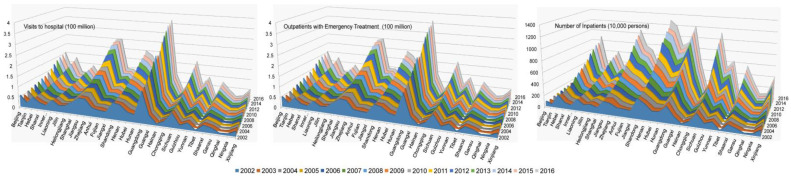
Main indicators for **RHC** in China.

Due to the limitations of the other variables, the eco-efficiency calculation period was determined to be 2002–2016. To reflect the **RHC** as comprehensively as possible, this paper tried to collect adequate data from various medical institutions (mainly from hospitals) from three indicators, which were selected as the dependent variables for Sensitivity Evaluation with Support Vector Machines (**SE-SVM**). Related indicators were the **VTH, OWT,** and **NOI** for the years from 2002 to 2016 (see as in Figure 1). These variables originate from the beginning of the Chinese Medical Health Statistics Yearbook in 2013. To achieve our goal, four aspects that are related to climate change (temperature, humidity, precipitation, and sunshine) and **REE** are the main independents variables, with other six controlling variables being considered in the following regression. The related data are from the China Statistical Yearbook and the Chinese Medical Health Statistics Yearbook for the years from 2001 to 2018.

### 2.2. Model Building

#### Sensitivity Evaluation with Support Vector Machines

Support Vector Machines (SVM) take the “Structural Risk Minimization Principle” as their main principle, which is in contrast to other computational learning theories. Through this method, it has been demonstrated that this method holds significantly improved advantages in terms of excellent classification ability and computing power [29]. Moreover, SVM-related methods are able to tackle practical problems that are easily affected by multiple factors, nonlinear relationships, and more complex data patterns [2,30,31]. In detail, Least Square-Support Vector Regression (**LS-SVR**) draws support from the kernel function (nonlinear) to map the data into a different high-dimensional space [27]. Linear learning machines learn linear relationships in a high-dimensional feature space [27], which is determined by a parameter that is irrelevant to the spatial dimensionality [32]. Therefore, LS-SVR is able to handle the multiple factors, regardless of whether they have a nonlinear or complex relationship.

Similar to basic Support Vector Machines [33], LS-SVR can be expressed by the following equation [32]:

(1) $$f\left(x|w\right)=y\left(x\right)=\overset{N}{\underset{k=1}{\sum }}\;\alpha_{k}K\left(x,x_{k}\right)+b$$

To achieve the goal, **SE-SVM** is used to combine LS-SVR-DS [34] with the Sensitivity Evaluation through a quickly implemented computer program [32].

(2) $$y\left(X;\sigma ,\gamma \right)=\overset{m}{\underset{i=1}{\sum }}\alpha_{i}\left(\sigma ,\gamma \right)K\left(x_{j},x_{i}\right)+b\left(\sigma ,\gamma \right)$$

Then the optimal parameters that are needed to minimize the average of squared errors are obtained [35] by the Equation (3). That is,

(3) $$\underset{\sigma ,\gamma }{\min}G\left(\sigma ,\gamma \right)=\frac{1}{m}\overset{m}{\underset{j=1}{\sum }}{\left[y_{j}-y\left(x_{j};\sigma ,\gamma \right)\right]}^{2}=\frac{1}{m}\overset{m}{\underset{j=1}{\sum }}{\left[y_{j}-\overset{m}{\underset{i=1}{\sum }}\alpha_{i}\left(\sigma ,\gamma \right)K\left(x_{j},x_{i}\right)-b\left(\sigma ,\gamma \right)\right]}^{2}$$

(4) $$\alpha =A^{-1}\left(y-bl_{v}\right)\;b=\frac{l_{v}^{T}A^{-1}y}{l_{v}^{T}A^{-1}l_{v}}$$

(5) $$K\left(x,x_{k}\right)=exp\left(-∥x-x_{k}{∥}^{2}/2\sigma^{2}\right)$$

$\alpha_{k}$ and b depend on γ and the kernels. $A=\Omega +\gamma^{-1}I$, t is a positive definite symmetric matrix for all γ>0, and $A^{-1}$ exists. **SE-SVM** adopts the RBF kernel to describe potential uncertain nonlinearity [30]. To integrate direct search into the LS-SVR, we have illustrated the search procedure below.

Step 1. Begin and initialize with a search point $B_{0}=(\sigma_{0},\gamma_{0})$ and k=1.

Step 2. Take the $B_{1}=(\sigma_{0}+\lambda_{\sigma },\gamma_{0}+\lambda_{\gamma })$ as an alternative point; $\lambda_{\sigma }$ and $\lambda_{\gamma }$ are the random step sizes originating from the uniform distribution of (0, 1).

Step 3. Compute $G\left(\sigma_{0},\gamma_{0}\right)$ and $G\left(\sigma_{0}+\lambda_{\sigma },\gamma_{0}+\lambda_{\gamma }\right)$ using (4) and (5).

Step 4. Update $\sigma_{0}$ with $\sigma_{0}+\lambda_{\sigma }$ and $\gamma_{0}$ by $\gamma_{0}+\lambda_{\gamma }$ if $G\left(\sigma_{0}+\lambda_{\sigma },\gamma_{0}+\lambda_{\gamma }\right)\leq G\left(\sigma_{0},\gamma_{0}\right)$. Otherwise, $\sigma_{0}=\sigma_{0}$ and $\gamma_{0}=\gamma_{0}$.

Step 5. If $G\left(\sigma_{0},\gamma_{0}\right)\leq \varepsilon$ or k≥N, stop the iteration. Otherwise, let k≥k+1 and go to Step 2. The iteration stops when either a desired accuracy is achieved or when the number of iterations exceeds a prespecified limit N. After the algorithm stops, this paper obtained the “optimal” pair of $\left(\sigma_{0},\gamma_{0}\right)$ for LS-SVR, which could be used to minimize training errors. Together, all of the above steps for the **SE-SVM** are used for sample learning and compare the prediction accuracy to judge the fit of the model by incorporating adequate information from the original data.

On basis of the steps above, the elasticity analysis method is introduced into the above algorithm. It can be considered that when the independent variable changes by one percent then that is how many percentage points the dependent variable also changes by. This new method is known as Sensitivity Evaluation with Support Vector Machines (**SE-SVM**). Steps 6 to 9 are the main procedures and operating instructions that will be used in the following empirical section:

Step 6. Learn and obtain the best parameters through the training samples and acquire the optimal parameter pair $\left(\sigma^{*},\gamma^{*}\right)$ using the LS-SVR-DS.

Step 7. Predict $\hat{\;y}\left(x_{\alpha 1}\right)$, $\hat{y}\left(x_{a2}\right)$, $\hat{y}\left(x_{\alpha 3}\right)$ …, and $\hat{y}\left(x_{\alpha n}\right)$ based on the optimal parameters of the **SE-SVM** model, where $x_{\alpha 1}=x_{1}*\left(1+1\%\right)$, $x_{\alpha 2}=x_{2}\left(1+1\%\right),\;x_{\alpha 3}=x_{3}\left(1+1\%\right)$, …, and $x_{n}\left(1+1\%\right)$.

Step 8. Compare $\hat{\;y}\left(x_{\alpha 1}\right)$, $\hat{\;y}\left(x_{\alpha 2}\right)$, …, and $\hat{y}\left(x_{\alpha n}\right)$ separately to obtain the quantitative evidence of the change degree for the temperature parameter from the climate change variable affecting the **RHC** when the **REE** increases in various regions across China.

Step 9. Then, repeat the step 6 to 8 for humidity, precipitation, and sunshine and explain each specific condition.

Step 10. Considering the regional characteristics, repeat all of the above steps and compute the related results for each spatial area until the corresponding model finds a satisfactory result and then stop the procedure. At this stage, the SE-SVM has finished the elastic analysis while using real data. This represents a novel way to analyze the interaction between variables by fusing machine learning and elastic concepts.

### 2.3. Parameter Setting

During the empirical portion of the process, SE-SVM can be set as the following parts: The empirical analysis comprises one main dependent variable and eight independent variables. Take y as the symbol for **RHC**. **VTH**, **OWT,** and **NOI, and** their relationship with **RHC** should be calculated separately. Meanwhile, $x_{1},x_{2},x_{3}$, …, and $x_{8}$ represent e Temperature, Humidity, Precipitation, Sunshine, and **REE** and controlling indicators such as **GDPPC,** **UL,** **PD,** **MP, LAD,** and **NHCI** (See as in Table 1) [32].

With the help of Steps 1 to 5, the first part forecasts the RHC using climate change data with the REE in China; in other words, **SE-SVM** is used along with the panel data from 2002-2016 to find the optimal sample fitting for the models with different dependent and independent variables. **MPE** (Prediction Error of Mean Percentage Error), **MSE** (Mean Square of Prediction Error), and **SDE** (Standard Deviation of Prediction Error) measure the prediction accuracy. That is, it is better to obtain a model with high forecasting accuracy or lower prediction errors overall, which can be achieved by sample learning.

With the assistance of Steps 6 to 9, the second part measures the impact of each variable in China. That is to say, with the best fitting model as the basic model, this paper applied the regional province-level data obtained China from 2002-2016 to the **SE-SVM** and then conducted elastic analysis and results comparison, allowing relevant empirical results to be drawn.

## 3. Results

### 3.1. Statistical Descriptive Analysis

#### 3.1.1. Spatial Characteristics of Regional RHC in China

The spatial characteristics of the regional **RHC** in China are an important prerequisite for further quantitative analysis, especially in China, which has a huge territory area. Although regional **RHC** are affected by local geological conditions, natural resources, customs, economic development, population density, cultural and educational background factors, etc., it is important to compare the spatial characteristics that are subject to geographic location, and climate change demonstrates the same spatial differences, meaning that these two aspects interact within a specific spatial range. China’s administrative divisions the principles of geographic isolation (natural barriers such as rivers, mountains) and geological geomorphology, which also determines the regional characteristics of climate change, take into account. Local residents are also subject to the unique impact of climate change in a specific location.

**Figure 2:**
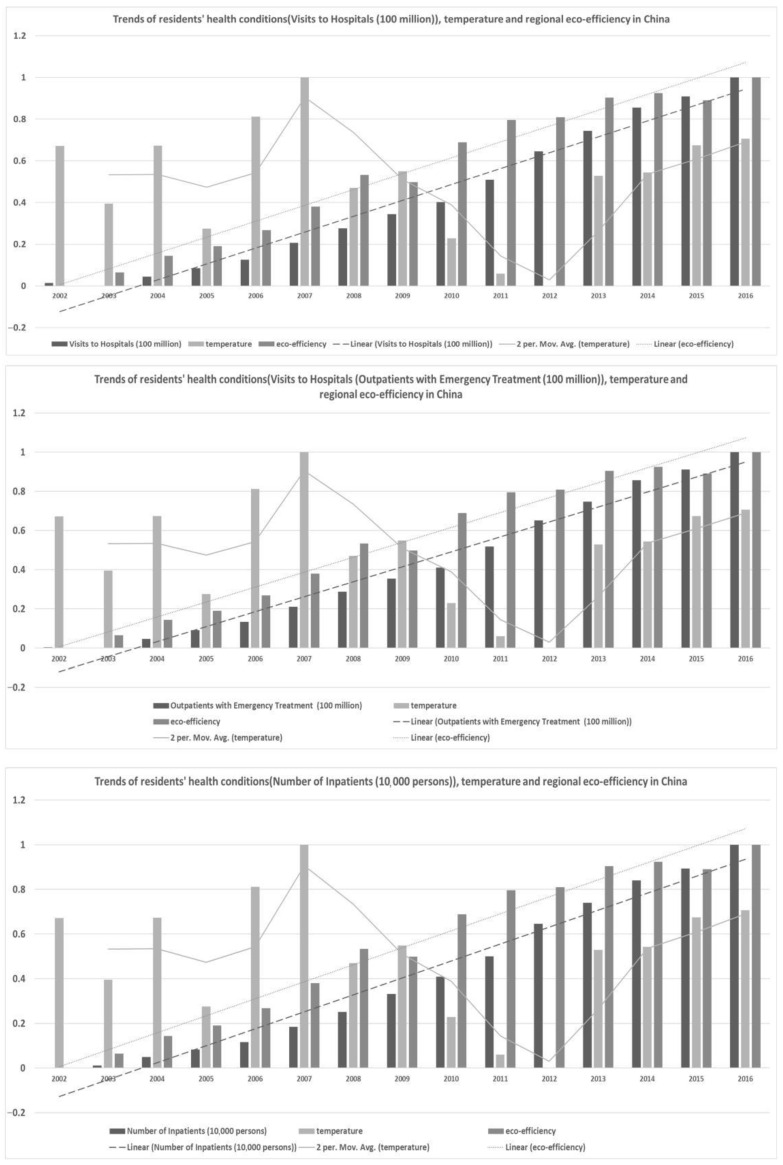
Trends of **RHC**, temperature, and **REE** in China.

According to original data from the 30 provinces and cities in China tat were selected for this study, Figure 2 provides three dimensions of VTH, OWT, and NOI to describe the **RHC** for different regions in China. As we can see, the three three-dimensional spatial distribution diagrams of VTH, OWT, and NOI exhibit almost the same characteristics as those for regional distribution, showing similar trends. Guangdong, Jiangsu, Zhejiang, Shandong, Henan, Sichuan, and Shaanxi are ranked at the forefront of China’s current and more urgent medical conditions. No matter the VTH, OWT, and NOI, each of them show a higher amplitude from 2002 to 2016. Some provinces show greater medical needs and pressure on **RHC**. The changes in the relevant characteristics and trends are crucial for formulating regional medical policies and for supplementing medical personnel and supplies. In the face of sudden medical and health incidents, the above problems are more urgent.

Furthermore, the spatial characteristics of the regional **RHC** in China force the proposed model to be computed by actual local conditions, which were denoted by the six control variables. Considering the regional differences and the implicit relationship between **RHC** and climate change, Figure 2 also shows the necessity of constructing a specific model to compute how climate change affects **RHC** for the individual provincial and municipal regions in China. It was shown that their trends were similar to each other and that the curves of **VTH, OWT,** and **NOI** were quite consistent and regionally different.

#### 3.1.2. Trend Analysis of Indicators in China

**Figure 3:**
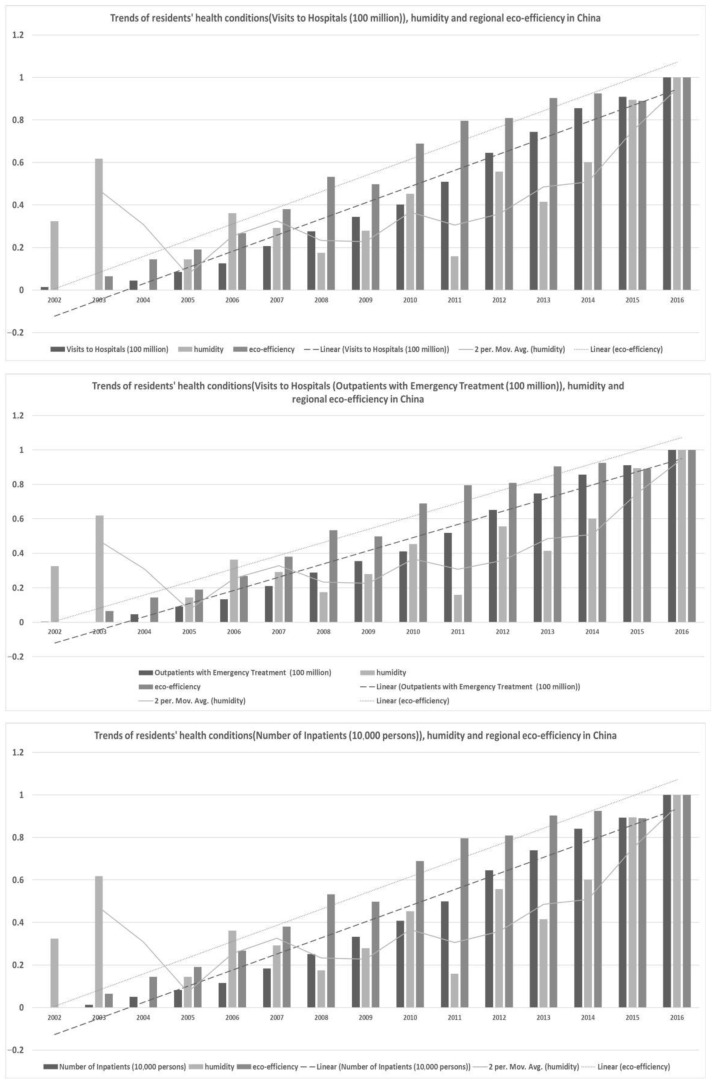
Trends of **RHC**, humidity, and **REE** in China.

**Figure 4:**
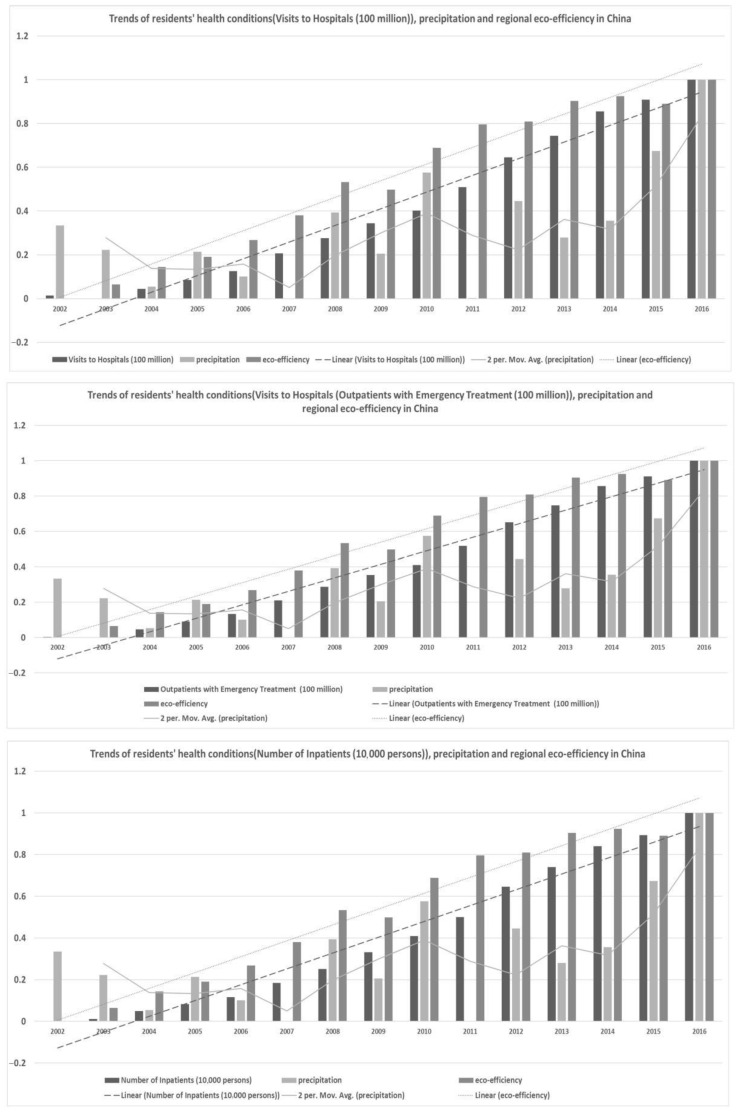
Trends of **RHC**, precipitation, and **REE** in China.

**Figure 5:**
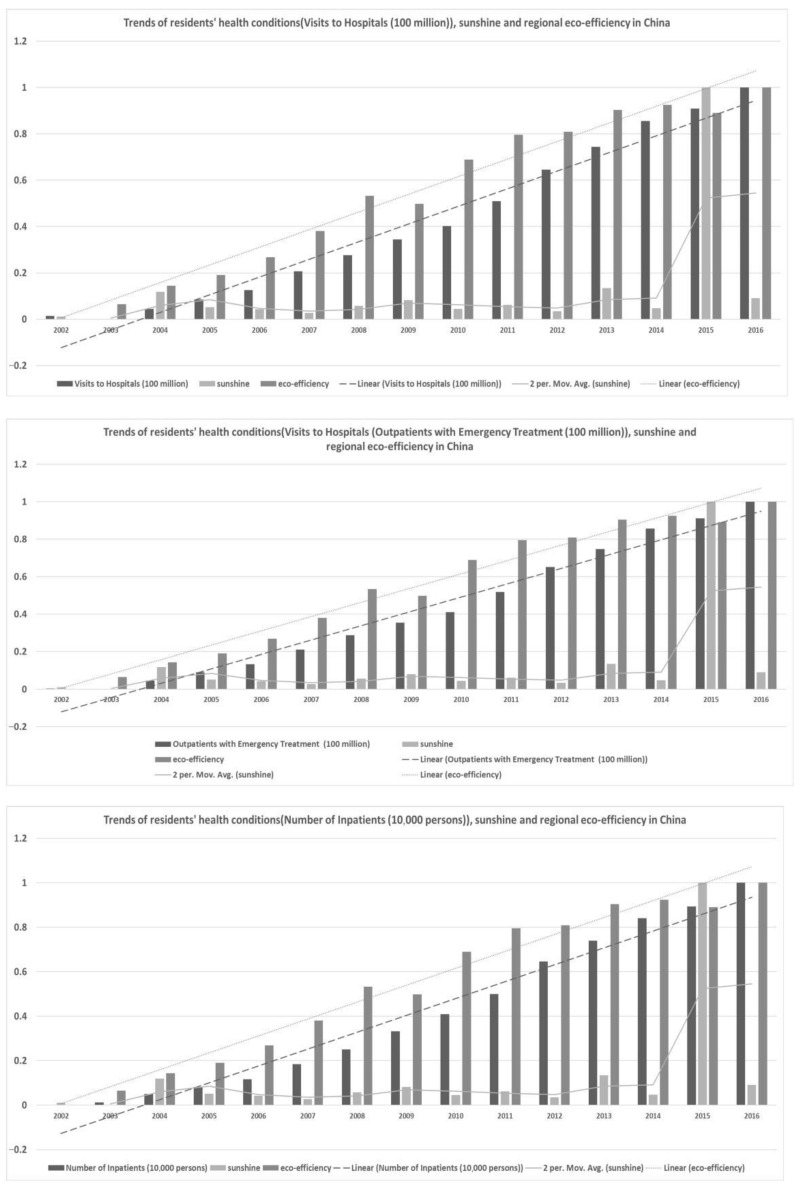
Trends of **RHC**, sunshine, and **REE** in China.

It shown that the tendency curves between the **RHC** and **REE** and one of the **temperature, humidity, precipitation, and sunshine** curves generally displayed a synchronous development trend. These findings confirm those from previous research. These findings also provided direct support for the empirical settings of **SE-SVM**. In detail, Figure 2, Figure 3, Figure 4 and Figure 5 show the trends of **RHC**, climate change, and **REE** in China. The three indicators **VTH, OWT, and NOI** and four different factors of climate change, **temperature, humidity, precipitation, and sunshine,** were compared in each graph in the following four figures. To reduce the impact of the size and dimension of the statistical units, the indicators were normalized and standardized. Three linear or moving average tendency lines were depicted above the real bar data. In each graph, the original values of the three **RHC** variables are represented by dark, medium, and light black shading. Their corresponding trend lines are represented by dotted, discrete, segmented, and straight lines. The following findings can be drawn:

First, **temperature, humidity, precipitation, and sunshine** experienced much larger fluctuations than **RHC did**, while **REE** shows an increasing trend line. There is a turning point for temperature in the years of 2007 and 2012, while sunshine displayed a sharp rising trend from 2014 to 2016. Additionally, this brings challenges to the model construction and quantitative calculations presented in this paper.

Second, **REE** and **RHC** remained relatively consistent and showed steady increases. The increase in eco-efficiency seems to maintain a similar level as the growing need for public health. This contradicts the idea that improving **REE** improves the energy use efficiency and that it is more conducive for the sustainable development of the environment. Therefore, this excellent correlation helps the interpretability of the model in this paper, but it real data analysis needs to be used in order to specifically analyze the degree of influence and the positive and negative directions of the two.

Third, there are significant differences in the nationwide change trends among the three variables of climate change, resident health conditions, and the improving **REE** in each figure. For each province and city in China, more attention should be paid to regional heterogeneity. On the one hand, it is helpful to add regional-wide control variables; on the one hand, it is necessary to construct specific impact measurement models for individual provinces or cities.

**Figure 6:**
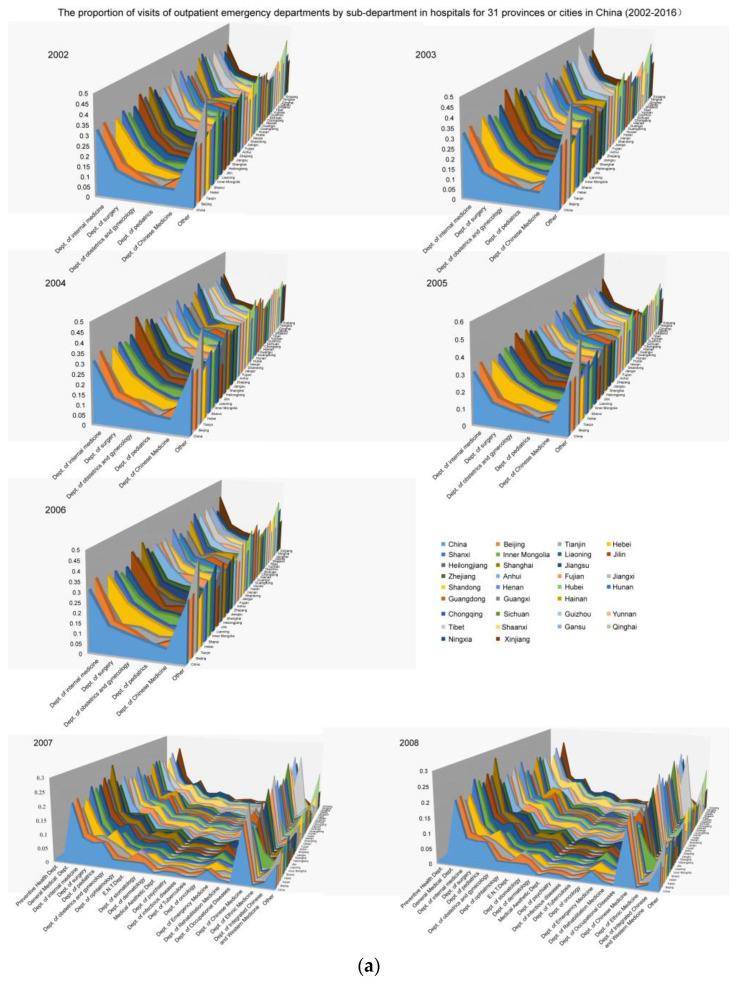

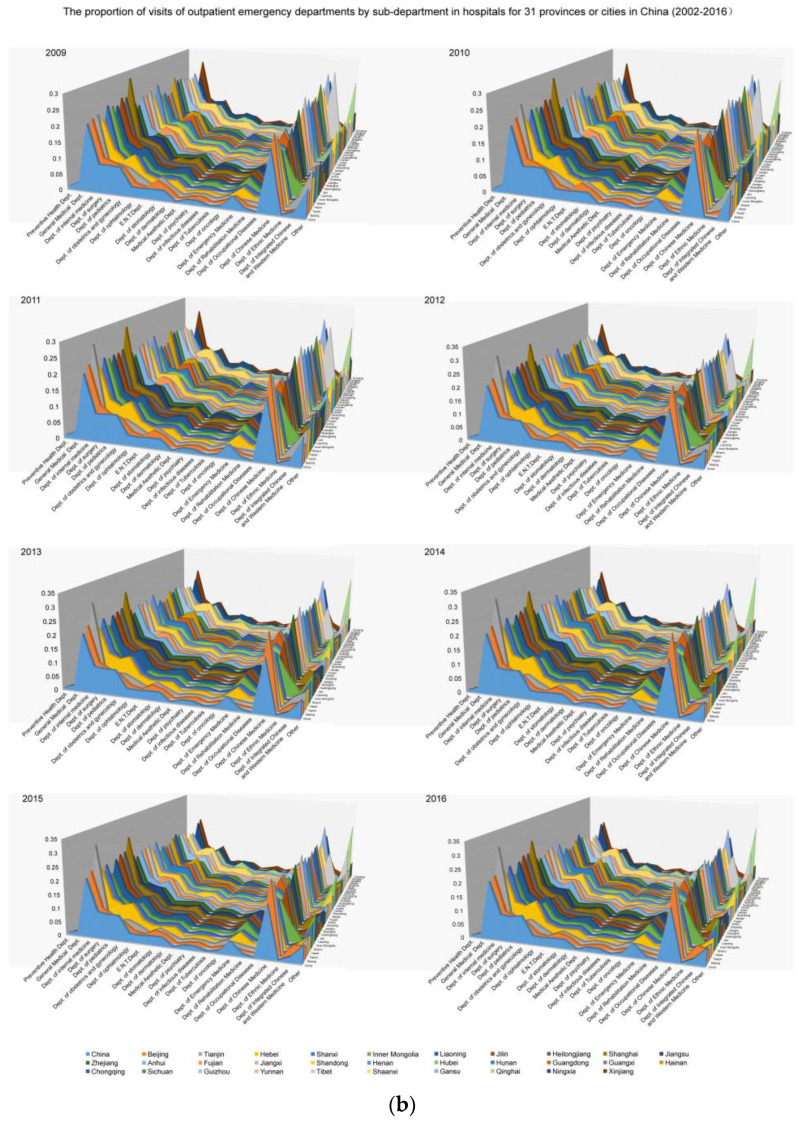
The number of outpatient emergency department visits to different sub-departments in China.

In addition, more microscopic evidence suggests that regional heterogeneity requires calculating the impact in each region in China. For example, by analyzing the data on visits to different sub-departments (such as pediatrics, internal medicine, Chinese Medicine, surgery, obstetrics and gynecology, and so on) in medical institutions, it is easier to determine that the different health problems that are targeted by different departments are closely related to climate change (see as Figure 6a,b). At the same time, social issues and government policies such as labor employment, fertility policies, and aging phenomena in the regional reality are important dimensions that can be used to explain such differences. Many scholars have recognized and supported the above-mentioned idea that climate change exhibits a crucial influence on the appearance of various diseases and that the reduction of environmental pollution by implementing eco-efficiency has an impact on public health.

### 3.2. Sample Learning and Prediction Accuracy Comparison

Comparing sample learning and prediction accuracy is necessary to forecast **RHC** using climate change data with the evidence of the improving **REE** in China. To gain a full understanding of the performance of the proposed method and detailed information regarding the basic variation that is the result of the effects of climate change on **RHC**, each of the following sections apply average temperature, average relative humidity, precipitation, and sunshine Hours separately and all of the above four indicators as the proxy variables for climate change in sample learning and forecasting for each year from 2002 to 2016. Then, the results with the best prediction accuracy for all three parts will be selected as the best model for elasticity analysis and will be used for further analysis.

$MPE=\frac{\sum_{t=1}^{T}\frac{y_{t}-\hat{y_{t}}}{y_{t}}}{T}$. $MSE=\frac{\sum_{t=1}^{T}{\left(y_{t}-\hat{y_{t}}\right)}^{2}}{T}$, $SDE=\sqrt{\frac{\sum_{t=1}^{T}{\left(y_{t}-\hat{y_{t}}\right)}^{2}}{T}}$. $y_{T}$ and $y_{t}$ denote the given sample value of the last year and the t. year. $\hat{y_{T}}$ and $\hat{y_{t}}$ denote the forecasted sample value for the last year and t year by LS-SVR. T=1,2,⋯,T. The combination of **MPE**, **MSE**, and **SDE** together is helpful to prove the advantages of **SE-SVM**, in which $y_{t}$ and $\hat{y_{t}}$ were forecasted for each year from 2002 to 2016 followed by the acceptable in-sample learning.

#### 3.2.1. Forecasting RHC Using Temperature with Improving REE

Most scholars hold that temperature is a main leading climate change index because the greenhouse gases that are generated by various human activities (especially economic development) and environmental changes have always been the focus of attention. average temperature is computed yearly and can represent average climate change directly as well as the effects on health conditions, as described in the Background Section.

Tables S1–S3 present the prediction accuracy comparison separately for the 30 provinces and regions that were considered in the present study. Additionally, the results in the tables show temperature as one of the four proxy variables of climate change and that it is a good forecaster or leading indicator that can be used to acquire information as it pertains to resident health conditions in advance, regardless of VTH, OWT, and NOI. At the level of the entire country, the MPE, MSE, and SDE of temperature to forecast VTH is as low as 0.000296, 0.005300, and 0.072800, respectively, and the averages of those values for the 30 provinces and cities are about 0.000616, 0.000298, and 0.009507, which is a quite high prediction accuracy, especially when considering the statistical unit of the predicted variable. the **MPE, MSE,** and **SDE** of temperature to forecast OWT is much lower, with values of about 0.000252, 0.004488, and 0.066990, the average level of those values for the 30 provinces and cities that were considered in the present study are very close, demonstrating values of about 0.000646, 0.000264, and 0.009109. However, when utilizing the temperature to forecast Number of Inpatients, the corresponding forecasting error became a little bigger than the former, with the **MSE** and **SDE** showing values of about 1970.296095 and 44.388017, although MPE is the lowest one, achieving a value of 0.000044.

**MSE** and **SDE** were better able to display the volatility and stability characteristics of the forecasting process when using **SE-SVM** in China. The tables show that the largest one appears when a utilizing the temperature to forecast Number of Inpatients, and this may be due to the fact that the influencing factors and functional relationships of hospitalized patients are more complicated. Considering climate change and the improvement of eco-efficiency and other regional characteristic variables is one aspect, and other potential factors (long-term living habits and impact accumulation) also need to be considered further in order to heighten the forecasting robustness. In additions, incorporating **REE** and various control variables in **SE-SVM** improved the interpretability and ensured the ability of the sample to be learned as well as its prediction accuracy.

#### 3.2.2. Forecasting RHC Using Humidity with Improving REE

The most beneficial humidity range for human health is 45% to 60%. If the air humidity is less than 45%, then it will cause indoor dryness, resulting in dry skin, throat, and respiratory tract, which may lead to asthma and other respiratory diseases. When the air humidity is higher than 60%, the human body will feel sluggish and uncomfortable. When the air humidity is higher than 80%, then the humidity is too high, making it difficult for the body to dissipate heat, resulting in symptoms such as increased body temperature, rapid heartbeat, dizziness, and nausea. Correspondingly, **RHC** will be affected by humidity. The average relative humidity is calculated yearly and can represent climate change directly and can also affect health conditions, as described in the literature review in Section 2.

Tables S4–S6 present the prediction accuracy comparison with sample learning data that were used for the **SE-SVM** for China as a whole as well as for the 30 selected provinces and cities that were used in this analysis. Additionally, the results in the tables show that humidity is one of the four proxy variables of climate change and that it is a good forecaster and leading indicator that can be used to acquire information regarding resident health conditions in advance, regardless of the Visits to Hospitals, OWT, and Number of Inpatients. For the whole country, the **MPE, MSE,** and **SDE** of humidity to forecast VTH are as low as 0.000259, 0.004053, and 0.063664, the average levels of those variables for the 30 provinces and cities that were included in this study were about 0.000495, 0.000208, and 0.008522, demonstrating a prediction accuracy that is quite high, especially when considering the statistical unit of the predicted variable. The **MPE** and **MSE** of the humidity to forecast OWT is much bigger, with values of about 0.003374 and 0.058088, and a lower SDE of 0.003374; the average level for the 30 provinces and cities that were selected for analysis in this study is much lower, with the **MPE being** 0.000219 and the **SDE** being 0.000219; however, a larger **MSE of** 0.008612 is also observed. However, when utilizing the humidity to forecast the Number of Inpatients, the corresponding forecasting error became a little bigger than the former results, with the **MSE** and **SDE** being shown to be 110.953421 and 6.908155, respectively, although the **MPE** is quite low, with a value of about 0.001227 being observed.

When using the temperature to forecast the Number of Inpatients, it demonstrated there was the best volatility and stability characteristics of the forecasting process of **SE-SVM** in China, it shown that humidity was the best variable to forecast the Number of Inpatients and that there is a similar explanation. Similarly, incorporating **REE** and various control variables improves the interpretability of the model and ensures learning and the prediction accuracy within the sample. The powerful learning ability and the effective support vectors help to describe the most suitable functional relationship through the kernel functions along with the addition of the regional heterogeneity for a single province or city in China.

#### 3.2.3. Forecasting RHC Using Precipitation with Improving REE

More varied precipitation from rainfall patterns may affect freshwater supply. A lack of safe water affects personal hygiene and increases the risk of diarrhea. The frequency and severity of floods are also rising. Floods contaminate the fresh water supply, increasing the risk of water-borne diseases and forming breeding grounds for disease-carrying insects such as mosquitoes. Floods can also cause drowning and bodily harm, destroy homes, and disrupt the supply of medical and health services. Average precipitation is calculated yearly and can represent climate change and affect health conditions directly, as described in the literature review in Section 2.

Tables S7–S9 present the prediction accuracy comparison with of the sample learning data for the **SE-SVM** for China as a whole and for each of the selected 30 provinces and cities. Additionally, the results in the tables show that precipitation is appropriate for use as one of the four proxy variables of climate change because it is a good forecaster and leading indicator that can be used to acquire the information regarding resident health conditions in advance, regardless of the number of Visits to Hospitals, OWT, and Number of Inpatients. From the whole country level, the **MPE, MSE,** and **SDE** of precipitation to forecast VTH are as low as 0.000296, 0.004604, and 0.067855; the average level of these values for the 30 provinces and cities is about 0.000638, 0.000273, and 0.010273, respectively, demonstrating a prediction accuracy that is quite high, especially when considering the statistical unit of the predicted variable. The **MPE, MSE,** and **SDE** of precipitation to forecast **OWT** is much lower, with values of 0.000252, 0.003812, and 0.061742 being obtained; the average level of these values for the 30 provinces and cities is also much lower, demonstrating values of about 0.000492, 0.000219, and 0.008830. However, similar to the ability of the temperature and humidity to forecast the Number of Inpatients and considering the characteristics of the volatility and stability of the forecasting process of the **SE-SVM** in China, it shown that the largest prediction errors appear when utilizing the humidity to forecast Number of Inpatients, which can be explained in a similar fashion as before.

#### 3.2.4. Forecasting RHC Using Sunshine with Improving REE

It is well-known that practitioners and researchers have reached a consensus on the influence of sunshine on people’s mood, brain, sleep, bones, and other aspects. Tables S10–S12 present the prediction accuracy comparison with the sample learning data from the **SE-SVM** for China as a nation and each of the 30 provinces and cities. Additionally, the results in the tables show that sunshine is appropriate for use as one of the four proxy variables of climate change because it is a good forecaster and leading indicator that can be used to for acquire information about resident health conditions in advance, regardless of the Visits to Hospitals, **OWT**, and Number of Inpatients. From the perspective of the whole country, the **MPE, MSE,** and **SDE** of sunshine to forecast **VTH** are as low as 0.000165, 0.001513, and 0.038903, and the average level of those values for the 30 provinces and cities is about 0.000461, 0.000158, and 0.007917, demonstrating a prediction accuracy that is quite high, especially when considering the statistical unit of the predicted variable. the MPE, MSE, and SDE of sunshine to forecast OWT is much lower, showing values of 0.000146, 0.001499, and 0.038719, with the average level of these values for the 30 provinces or cities is also much lower, showing values of about 0.000513(bigger than 0.000461), 0.000141, and 0.007565. These main findings are all acceptable.

However, it seems that similar to the temperature, humidity, and precipitation, the largest prediction errors appear when utilizing the humidity to forecast the Number of Inpatients, especially when considering the characteristics of the volatility and stability of the **SE-SVM** forecasting process in China. Therefore, forecasting the Number of Inpatients for the **RHC** does not perform as well as any of the other variables, but the individual and average forecasting accuracy for China as a whole and for each province and city is acceptable, while the fluctuations seen in the prediction accuracy are relatively large.

#### 3.2.5. Forecasting RHC Using Four Factors with Improving REE

In fact, the four climate change indicators generally affect the health of residents at the same time, together with the economic and environmental changes that are caused by promoting higher regional eco-efficiency. Therefore, it became more realistic to consider corresponding specific comprehensive impacts simultaneously. Tables S13–S15 present the prediction accuracy comparison with the sample learning data for the **SE-SVM** for China as a whole and for each of the 30 provinces and cities. Additionally, the results in the tables show that in terms of the four climate change factors together, they represent good forecasters and leading indicators that can be used to acquire resident health condition information in advance, regardless of the Visits to Hospitals, **OWT**, and Number of Inpatients. Additionally, the largest prediction errors appear when utilizing the humidity to forecast Number of Inpatients when considering the volatility and stability characteristics of the **SE-SVM** forecasting process in China.

Together, Table 2, Table 3 and Table 4 summarize the findings and show that the forecasting difference for each of the three **RHC** indicators using different model settings and four different indicators: **SE-SVM** with temperature, **SE-SVM** with humidity, **SE-SVM** with precipitation, and **SE-SVM** with sunshine and **SE-SVM**. These results demonstrate results that are similar to those that have been obtained in the past: (1) Climate change is a good predictor for predicting resident health, as it takes into account improved **REE** and other control variables that reflect regional heterogeneity; (2) better model settings can be found by comparing the average **MPE, MSE,** and **SDE** for temperature, humidity, precipitation, sunshine together; (3) considering the real situation where temperature, humidity, precipitation, and sunshine interact, we chose the **SE-SVM** with four indicators to be the basic model for elastic analysis and also incorporated the improving eco-efficiency and other control variables.

### 3.3. Findings and Explanations

Based on the **SE-SVM** using the four indicators, this section measured the specific impact of climate change on resident health conditions by considering the improving **REE** in China. Table 5, Table 6 and Table 7 present how the visits change when each of the independent variables increases by 1% as each of the 11 dependent variables increase by 1%, with the phenomenon being denoted as X × (1 + 0.01).

When observing the nation as a whole, it can be concluded that temperature, humidity, precipitation, and sunshine have a positive relationship with **VTH** or **OWT** but a negative one with the NOH. In other words, when each indicator of change occurred or increased by 1%, then the **VTH** increased by 0.004451, 0.006776, 0.000462, and 0.001071 and the **OWT** increased by 0.004674, 0.006668, 0.001324, and 0.001614. Meanwhile, the NOH decreased by 0.006938, 0.002041, 0.001168, and 0.000561. The positive change relationship confirms the conclusion that climate change affects the health status of residents; that is, it increases the incidence of various diseases and the medical needs of the residents in all aspects. The negative change relationship shows that climate change has reduced some of the resident medical needs under certain conditions, which is contrary to our intuitive perception and requires more in-depth research in the future.

Further, it is obvious that when **REE** increases by 1%, then the rate of climate change will decrease by that same percentage, increasing the needs of VTH and OWT by 0.002242 and 0.002688 but decreasing the needs of NOH. The reason for this is that reducing the impact of polluted gas, water, and solid waste as a result of improved eco-efficiency on the health of residents is a long-term process, and most hospitalized patients are cases that have accumulated over a long period of time and are in serious condition. One reason for the negative impact of improving eco-efficiency on short-term outpatient and emergency services is that the environmental changes that can be made to eco-efficiency mostly affect public health through the climate change effects of four proxy factors. For example, carbon dioxide in waste gas is a greenhouse gas, and wastewater affects drinking water, food safety, and agricultural irrigation. It is worth paying attention to this aspect in the future longer-term research.

In addition, controlling variables such as **GDPPC, UL, PD, MP, LAD,** and **NHCI** have explained the corresponding changes in resident health conditions in terms of affordability, population impact, medical supply, quality of service, technical conditions, etc. The improvement of the above factors has increased the need for the diagnosis and treatment of resident health conditions to a certain extent. This is because the scale medical institution and service development in China is far from being able to meet the growing needs of the country. At the same time, the aging society and the second-child policy have created new, more, and diversified medical needs, regardless of type, amount, and quality.

At the provincial level, there are indications that there are large differences among the 30 Chinese provinces and cities that were selected for the current analysis . Applying **SE-SVM** to various regions by means of actual data, it was found that the quantitative impact of the four proxy factors of climate change on **RHC** varies greatly, and the signs or values of eco-efficiency and other control variables are different too. However, roughly 60% of the provinces, cities, and regions agree with the nationwide conclusions. The model settings for the controlling variables reflected regional heterogeneity, which cannot be displayed in the model of the national-level data in China. The differences in the industrial structure, resource endowments, and related medical insurance policies in the development of regional economies are also one of the reasons for the above. Economically developed regions rely on high-tech industries with lower resource consumption and greater added value. The living environment is continuing to improve, the medical facilities are becoming more complete, and medical standards are higher. The values of the control variables are larger. Climate change combined with **REE** and **GDPPC, UL, PD, MP, LAD,** and **NHCI** are helpful to explain specific effects. Therefore, the main findings further emphasize the necessity of differentiating research in various regions in China. Each province or city should formulate a reasonable sustainable development strategy and medical policy based on its own real conditions to reduce the adverse aspects of climate change on local inhabitants.

## 4. Discussion

Climate change is closely associated with a variety of disease, and humans ask for professional assistance from local clinics, hospitals, or medical institutions to alleviate the negative effects that climate change has on their health. The pathways through which climate change affect health have been widely investigated [5]. The WHO has reported that the global temperature and precipitation have increased over past 30 years, and more than 150,000 lives have been lost because of the anthropogenic changes that are caused by climate change year after year. Moreover, the effects of climate change-induced trauma has also been investigated [15]. As a whole, it has been shown that climate change can be taken as one important explanation for the main **RHC** problems.

There is no doubt that humans need suitable living conditions that consist of clean air, safe drinking water, healthy and sufficient food, secure shelter, among other qualities, and climate change poses a threat to many of these things. Meanwhile, sustainability development has forced humans to continue to improve **REE** [36], the main task of which is to minimize greenhouse gases as well as to reduce the amount of polluted air and water and solid waste [28]; in times, this process should also have a positive impact on public health. In the above context, it is an interesting issue to investigate how climate change is able to impact resident health conditions as the **REE** improves in China.

From another perspective, **REE** represents the results of short-term economic behavior and environmental changes, and climate change shows the long-term implicit variation trends for basic climate factors. Therefore, **REE** and climate change together are important leading and inseparable indicators, and it is necessary to discuss the specific impact of these two variables on RHC. This study provided empirical quantitative evidence for decision makers. The findings that were discussed here can serve as a basis for assessing the relationship between high-quality of economic development, environmental sustainability, and public health.

(1) The quantitative conclusions of the effects of climate change and health are of great help to human beings in mitigating the various negative effects of ongoing climate change and in improving public health. Additionally, they can enlighten scholars or decision makers to pay attention to the implicit changes and mechanisms of temperature, humidity, precipitation, and sunshine on public health from the wider perspective of biomedicine and life science. It is necessary to understand the relationship between climate change, public health, and sustainable activities, and it is very important for the government to understand the current status of the medical system and to improve the allocation of medical resources, especially when considering climate change and the improving **REE** in China.

(2) Obtaining effective data on public health and its existing problems in advance can result in manpower, space, equipment, and time being more flexible and can improve the existing foundation, especially when there is a medical emergency, as this effect is more significant.

Forecasting the **RHC** by means of **SE-SMM** has created the possibility of improving the quality of medical services and the effects of these improvements on the health of residents. For instance, when a large-scale influenza pandemic (such as the 2020 COVID-19 pandemic) spreads, the number of outpatients in hospitals surges, with masks and disinfectants becoming important resources for the protection of the health of residents, and the coordination between provinces and cities and the shortage of epidemic areas have become the primary problems that remain to be solved. Without prior judgment and preparation, such diseases will therefore likely affect the lives of residents as governments attempt to control epidemic spread before an effective vaccine is successfully developed. In addition, in the daily operation of medical institutions and the daily maintenance of resident health, the prediction of short-term demand changes and long-term demand trends for different sub-department’s helps doctors’ work arrangements, the safety of the technical equipment supply chains, and the reasonable allocation of medical resources.

(3) From another perspective, the elastic analysis findings as well as the regional characteristics and existing factor endowments (represent by **GDPPC, UL, PD, MP, LAD,** and **NHCI**) help to understand public health problems in more depth. Variables such as basic medical level directly inform us of the medical gap between regions and the complex needs that are caused by climate change. Table 5, Table 6 and Table 7 gave the specific magnitude and direction of other aspects caused by changes in the control variables, allowing decision makers in different regions to specify differentiated response measures.

## 5. Conclusions

Research on the relationship between climate, health, and economic activity is one of the most popular areas of interdisciplinary research, but research rarely focus on their quantitative relationship from a spatial provincial level. Therefore, according to the reality and previous literature on the relationship between public health, climate change, and the economic activities that promote sustainability, this article aimed to obtain the macro-effects of climate change on resident health, including eco-efficiency (representing economic activities that emphasize environmental sustainability) and regional differences. At the same time, selecting machine learning methods to construct **SE-SVM** helped to obtain more accurate empirical results. During the empirical process, the **SE-SVM** was applied to Chinese provincial panel data, taking the Visits to Hospitals, Outpatients with Emergency Treatment, and Number of Inpatients as proxy variables for resident health conditions, and temperature, humidity, precipitation, and sunshine were used as climate change variables, simultaneously incorporating the calculated eco-efficiency with six controlling indicators.

The main empirical findings can be summarized as the following three aspects:

First, climate change could serve as a forecaster for resident health conditions, it was shown that (1) each climate change indicator–temperature, humidity, precipitation, and sunshine—was a good predictor for resident health, especially when taking improved **REE** and other control variables into account; (2) the four climate change indictors together served to be a good predictor; (3) the variation of the **NOH** prediction error was much bigger than the prediction errors for the **VTH and OWT** in all of the forecasting models, regardless of whether temperature, humidity, precipitation, sunshine or the four indicators together were being used; (4) incorporating **REE** and various control variables reflecting regional heterogeneity improved the interpretability of the model and ensured learning within the sample and the prediction accuracy; (5) **SE-SVM** was helpful to describe the functional relationship through kernel functions, especially when regional heterogeneity was added for a single province and city, the results of which were better than those that were obtained the China as an entire nation.

Second, climate change affected the resident health conditions, indicating that (1) at a nation level, it could be concluded that temperature, humidity, precipitation, and sunshine had a positive relationship with **VTH** or **OWT** but a negative one with the NOH. (2) When the **REE** increased by 1%, then climate change reduced by that same percentage, causing the needs of **VTH OWT** to increase by 0.002242 and 0.002688 but decreasing the needs of **NOH**; (3) the impact of climate change on **RHC** varied greatly, and the effects of eco-efficiency and the control variables were also inconsistent.

Third, regional heterogeneity was present for both individual provinces and individual cities, but the increasing regional eco-efficiency did not always demonstrate positive effects in different regions, and a variety of local activities and regional natural resource factors were potentially important explanatory factors for this.

Quantitative impact analysis is helpful for optimizing policy formulation and implementation measures for economic, environmental, and public health cross domains. For example, such analyses can act as a reference for detecting the impact of climate change on health, optimizing medical resources in advance to meet the needs of different regions and rationally evaluating the effects that an improved eco-efficiency has on humans, etc.

In future research, improvements in the following areas will be conducive to the deepening of the research in this article. First, it will be important to analyze the effects of climate change on different types of health problems. Second, considering the influence of variables such as the psychological conditions of residents as well as their living habits is another direction for future research. Third, attention should be paid as to how to obtain more bigger and micro real-time data and appropriate methods to portray climate change and public health.

## Figures and Tables

**Table 1:** Variable name and abbreviation.

Names	Abbreviation	Proxy Variable
Climate Change	**CC**	Temperature, humidity, precipitation, and sunshine
Residents’ Health Condition	**RHC**	the Visits to Hospitals (VTH), Outpatients with Emergency Treatment (OWT), and Number of Inpatients (NOI)
Regional eco-efficiency	**REE**	Computed by the specific method
GDP per capita	**GDPPC**	Computed by the specific method
Urbanization level	**UL**	Computed by the specific method
Population density	**PD**	Computed by the specific method
Medical Personnel	**MP**	Computed by the specific method
Licensed (Assistant) Doctors	**LAD**	Computed by the specific method
Number of health care institutions	**NHCI**	Computed by the specific method

**Table 2:** Forecasting the VTH using different model settings.

Model with Different Variables	MPE	MSE	SDE
	Average Value		
SE-SVM with temperature	0.000616	0.000298	0.009507
SE-SVM with humidity	0.000461	0.000158	0.007917
SE-SVM with precipitation	0.000638	0.000273	0.010273
SE-SVM with sunshine	0.000495	0.000208	0.008522
SE-SVM with four indicators	0.000585	0.000564	0.011054

**Table 3:** Forecasting OWT using different model settings.

Model with Different Variables	MPE	MSE	SDE
	Average Value		
SE-SVM with temperature	0.000646	0.000264	0.009109
SE-SVM with humidity	0.000513	0.000141	0.007565
SE-SVM with precipitation	0.000492	0.000219	0.008830
SE-SVM with sunshine	0.000584	0.000219	0.008612
SE-SVM with four indicators	0.000652	0.000520	0.009997

**Table 4:** Forecasting NOH using different model settings.

Model with Different Variables	MPE	MSE	SDE
	Average Value		
**SE-SVM** with temperature	0.000657	107.4898	6.287633
**SE-SVM** with humidity	0.001420	60.32005	5.895260
**SE-SVM** with precipitation	0.001214	96.56187	6.108994
**SE-SVM** with sunshine	0.001227	110.9534	6.908155
**SE-SVM** with four indicators	0.001610	41.50525	4.463848

**Table 5:** How the **VTH** change when each of variables increases by 1% as X × (1 + 0.01).

Regions	Temperature	Humidity	Precipitation	Sunshine	REE	GDPPC	UL	PD	MP	LAD	NHCI
China	0.004451	0.006776	0.000462	0.001071	0.002242	0.002969	0.003596	0.003265	0.003350	0.004602	0.000805
Beijing	0.001283	0.004197	0.000860	−0.007015	0.002101	0.002258	0.005336	0.003885	0.003301	0.003762	0.000832
Tianjin	0.002517	−0.001033	−0.000725	−0.010497	0.002187	0.000887	0.002094	0.003399	0.002424	0.004436	0.000504
Hebei	0.003451	−0.003348	−0.006325	−0.007646	−0.005947	−0.005510	−0.006628	0.008791	−0.004094	−0.001067	−0.007339
Shanxi	−0.007094	−0.001168	0.001367	−0.001913	−0.002430	0.004894	0.004166	0.008154	0.000552	0.008288	−0.001949
Inner Mongolia	0.000125	−0.003873	0.001058	−0.000213	−0.000002	0.002085	−0.000077	0.017445	0.017826	−0.002580	−0.004218
Liaoning	−0.002413	−0.007565	−0.004315	−0.003603	−0.004651	−0.004278	−0.000512	0.012959	−0.000632	0.000995	−0.004862
Jilin	−0.000749	−0.009097	0.000515	−0.003592	0.001879	0.001629	0.015148	0.041778	0.002624	0.003510	0.000678
Heilongjiang	−0.001493	−0.006132	0.000519	−0.000007	−0.000963	0.001690	0.018419	−0.009811	0.002846	0.003307	−0.000397
Shanghai	−0.023734	−0.003612	0.001341	0.004557	−0.000120	0.002950	0.003422	0.006900	0.002418	0.004339	0.000893
Jiangsu	0.020299	0.025642	0.021769	0.022824	0.022343	0.024143	0.024239	0.034792	0.026035	0.028391	0.022610
Zhejiang	0.000705	0.000266	0.005769	0.003770	0.007134	0.007221	0.009330	0.009098	0.007629	0.007634	0.006364
Anhui	−0.003214	0.004309	−0.001023	−0.002827	0.001341	0.002022	0.003393	−0.000016	0.001895	0.003397	0.000037
Fujian	−0.015584	−0.008090	0.001076	0.001022	−0.000495	0.004805	0.004830	−0.011098	0.003831	0.000417	−0.000543
Jiangxi	−0.010566	−0.010538	−0.013728	−0.014058	−0.012981	−0.011313	−0.010511	−0.005173	−0.011778	−0.008679	−0.013417
Shandong	−0.000641	−0.006621	−0.006215	−0.006554	−0.007207	−0.002889	−0.006825	0.017850	−0.003590	−0.003145	−0.007306
Henan	−0.002355	0.002674	−0.001243	0.001189	0.000539	0.002652	0.004119	−0.013767	0.002607	0.003349	−0.000468
Hubei	0.003503	−0.000548	−0.002005	−0.001008	0.001365	0.001798	−0.000829	0.019420	0.000692	0.001838	−0.000281
Hunan	0.003824	−0.003003	−0.002315	−0.004271	−0.000950	−0.001206	0.001620	−0.002697	−0.000659	0.000239	−0.002680
Guangdong	−0.008089	−0.003668	−0.000830	−0.001291	−0.001233	0.005320	0.000544	0.003087	0.007437	0.005590	−0.000805
Guangxi	0.005726	0.000785	0.000107	−0.001048	0.001212	0.001240	0.004103	0.005328	0.001429	0.002027	−0.000299
Hainan	0.005771	−0.004605	0.000211	−0.000009	0.001314	0.003484	0.000371	0.020602	0.003911	−0.000786	−0.000069
Chongqing	0.001143	−0.007133	−0.003717	−0.006084	−0.004602	−0.004155	−0.002879	0.011133	−0.003888	−0.003079	−0.005319
Sichuan	−0.003345	0.000084	−0.003471	−0.003232	−0.002882	−0.000874	−0.001889	0.019777	0.000034	0.001637	−0.003929
Guizhou	0.002775	−0.003198	0.000140	0.000468	0.002288	0.002423	0.002809	0.001113	0.002763	0.003174	0.000317
Yunnan	0.000476	0.001216	0.000405	−0.001650	0.000322	0.002494	0.009080	−0.008375	0.002955	−0.000429	0.000063
Shaanxi	−0.010195	−0.012365	−0.011844	−0.013620	−0.011054	−0.010285	−0.008617	0.026870	−0.009776	−0.007611	−0.011866
Gansu	−0.006756	−0.007688	−0.007109	−0.006825	−0.008234	−0.006450	−0.006203	0.031037	−0.005365	−0.002544	−0.007013
Qinghai	0.008481	0.008258	−0.000506	−0.000608	0.000214	0.001290	0.008204	0.018339	0.001777	−0.001900	0.000823
Ningxia	−0.007574	−0.011093	−0.009466	−0.010137	−0.008768	−0.008628	−0.006822	−0.002711	−0.007689	−0.006139	−0.009665
Xinjiang	−0.009132	−0.009686	−0.008010	−0.007312	−0.009156	−0.004829	−0.015593	0.003507	−0.004000	−0.003490	−0.009480

**Table 6:** How **OWT** change when each of variables increases by 1% X × (1 + 0.01).

Regions	Temperature	Humidity	Precipitation	Sunshine	REE	GDPPC	UL	PD	MP	LAD	NHCI
China	0.004674	0.006668	0.001324	0.001614	0.002688	0.003637	0.003903	0.003481	0.004095	0.005531	0.001218
Beijing	0.001201	0.003708	0.000201	−0.006614	0.001639	0.001729	0.005730	0.003198	0.002621	0.003057	0.000308
Tianjin	0.003158	−0.001127	−0.000610	−0.010642	0.002320	0.000882	0.002243	0.003537	0.002516	0.004336	0.000492
Hebei	0.002469	−0.004974	−0.007605	−0.008777	−0.006956	−0.006806	−0.007342	0.007331	−0.005513	−0.002740	−0.008415
Shanxi	−0.007090	−0.000260	0.001159	−0.001523	−0.002161	0.004727	0.005975	−0.000515	0.000389	0.008415	−0.001875
Inner Mongolia	0.000347	−0.003903	0.000857	−0.000757	−0.000123	0.001730	0.004797	0.005273	0.016834	−0.002339	−0.004267
Liaoning	−0.000047	−0.005065	−0.002237	−0.000901	−0.002542	−0.002202	0.001845	0.013767	0.001423	0.003224	−0.002795
Jilin	−0.002937	−0.010688	−0.001947	−0.003870	−0.000584	−0.000890	0.004504	0.010607	0.001089	0.000539	−0.001842
Heilongjiang	−0.002070	−0.006521	0.000236	−0.000002	−0.000915	0.001859	0.020543	−0.002407	0.002550	0.002930	−0.000380
Shanghai	−0.023560	−0.003453	0.001402	0.004552	−0.000096	0.002997	0.003338	0.007003	0.002366	0.004319	0.000889
Jiangsu	0.001003	0.001621	0.002217	0.002865	−0.003814	0.004013	−0.001140	0.064399	0.023800	0.000919	−0.000639
Zhejiang	0.002085	0.001685	0.006717	0.004781	0.008158	0.008198	0.010475	0.010401	0.008621	0.008651	0.007324
Anhui	−0.002322	0.004500	−0.001126	−0.002775	0.001410	0.002073	0.003657	0.000926	0.001577	0.003213	−0.000101
Fujian	−0.018382	−0.008644	0.001232	0.001205	−0.000461	0.003680	0.004510	0.002424	0.003138	0.001191	−0.000502
Jiangxi	0.007092	0.006016	−0.000260	−0.001584	0.000254	0.001011	0.002903	0.012770	0.001212	0.002394	−0.000208
Shandong	−0.001178	−0.007573	−0.005624	−0.005833	−0.007772	−0.001222	−0.006793	0.017512	−0.002567	−0.002525	−0.007231
Henan	−0.001659	0.003409	−0.001077	0.001603	0.000309	0.002797	0.004323	−0.008589	0.002794	0.003738	−0.000644
Hubei	0.003915	−0.000319	−0.001985	−0.000938	0.001465	0.001862	−0.002454	0.018168	0.001392	0.001443	−0.000418
Hunan	0.003894	−0.002936	−0.002054	−0.004032	−0.000701	−0.000945	0.001883	−0.002835	−0.000453	0.000339	−0.002443
Guangdong	−0.008472	−0.004059	−0.000857	−0.001445	−0.001096	0.005009	0.000297	0.001865	0.007394	0.006364	−0.000844
Guangxi	0.004430	0.000280	−0.000267	−0.001015	0.000734	0.000877	0.004902	0.003418	0.001252	0.001864	−0.000661
Hainan	−0.003015	−0.002534	−0.004858	−0.004893	−0.003830	−0.002409	−0.004592	0.007739	−0.001979	−0.002292	−0.004966
Chongqing	−0.000850	−0.007312	−0.004859	−0.007261	−0.005652	−0.005306	−0.003901	0.009229	−0.005036	−0.004245	−0.006472
Sichuan	−0.003727	−0.000189	−0.004070	−0.003960	−0.003262	−0.001676	−0.001928	0.020370	−0.001043	0.000356	−0.004372
Guizhou	0.004351	−0.001200	0.000321	0.000822	0.002359	0.002379	0.002926	−0.003810	0.002602	0.003088	0.000533
Yunnan	0.002103	0.002381	0.000590	−0.001367	0.000439	0.002931	0.007184	−0.001700	0.003835	−0.000951	0.000256
Shaanxi	−0.007703	−0.009974	−0.009624	−0.011401	−0.008786	−0.008077	−0.006291	0.032971	−0.007624	−0.005661	−0.009663
Gansu	−0.012006	−0.013069	−0.012174	−0.012346	−0.012984	−0.011571	−0.010535	0.029066	−0.010650	−0.008114	−0.011958
Qinghai	−0.029506	−0.027637	−0.033198	−0.030915	−0.031876	−0.031590	−0.029141	−0.020822	−0.031371	−0.030953	−0.032269
Ningxia	−0.007340	−0.010092	−0.008647	−0.009447	−0.008338	−0.007787	−0.006188	−0.002111	−0.006699	−0.004845	−0.008923
Xinjiang	−0.007802	−0.005777	−0.006053	−0.005065	−0.007182	−0.002259	−0.014325	0.012056	−0.002263	−0.004293	−0.007364

**Table 7:** How **NOH** change when each of variables increases by 1% as X × (1 + 0.01).

Regions	Temperature	Humidity	Precipitation	Sunshine	REE	GDPPC	UL	PD	MP	LAD	NHCI
China	−0.006938	−0.002041	−0.001168	−0.000561	−0.006272	0.004420	−0.001231	0.011291	0.008106	0.007393	−0.002325
Beijing	−0.007270	−0.010300	−0.010287	−0.008262	−0.006638	−0.007318	−0.010236	−0.007331	−0.006247	−0.005261	−0.010062
Tianjin	−0.009590	−0.014090	−0.012060	−0.012262	−0.011122	−0.010921	−0.009989	−0.009076	−0.008865	−0.008397	−0.012164
Hebei	−0.010210	−0.014472	−0.015701	−0.017101	−0.014527	−0.014553	−0.015717	−0.001216	−0.013776	−0.011240	−0.016344
Shanxi	−0.013030	−0.001747	−0.001872	−0.003113	−0.004805	0.000019	0.005479	0.010150	−0.001561	0.003628	−0.005537
Inner Mongolia	−0.001643	−0.001959	0.001582	−0.001329	−0.001110	0.005029	0.005594	0.025706	0.008492	0.001653	−0.002389
Liaoning	0.004100	−0.002911	0.001248	0.002624	0.000303	0.001253	0.008056	0.021466	0.006757	0.008906	0.000405
Jilin	−0.000194	−0.006381	0.000683	0.000076	0.002329	0.002326	0.012399	0.038181	0.005236	0.001987	0.000587
Heilongjiang	−0.002021	−0.004442	−0.000469	−0.000052	−0.001379	0.003801	0.023983	−0.031683	0.002878	0.005545	−0.000826
Shanghai	−0.007078	0.003055	0.002357	0.002537	0.002201	0.002900	0.005000	0.004302	0.002329	0.003563	0.000848
Jiangsu	−0.000340	0.008708	0.004004	0.004921	0.005081	0.006638	0.006976	0.014416	0.007750	0.010294	0.004385
Zhejiang	−0.000407	−0.001082	−0.000746	0.000619	−0.002026	0.001393	0.000359	−0.000997	0.024487	−0.011894	−0.001601
Anhui	−0.003063	0.000756	−0.002532	−0.003611	0.000084	0.000725	0.002307	0.001878	−0.000048	0.001799	−0.002012
Fujian	0.000763	0.010818	0.008843	0.009652	0.009503	0.012912	0.013735	0.019929	0.012659	0.012889	0.009804
Jiangxi	−0.014116	0.005288	−0.001032	−0.001441	0.000527	0.001287	0.003268	0.006610	0.001185	0.003235	0.000179
Shandong	−0.005908	−0.011717	−0.009656	−0.010409	−0.011700	−0.005725	−0.010247	0.023214	−0.005644	−0.007830	−0.011045
Henan	−0.003040	0.000028	−0.001667	0.002091	0.000525	0.003172	0.005048	−0.013862	0.003112	0.003872	−0.000479
Hubei	0.006674	0.008180	0.005239	0.006109	0.007677	0.007470	0.008380	0.042928	0.008395	0.009715	0.006356
Hunan	0.002323	−0.001690	0.000800	−0.001109	0.002248	0.002145	0.004854	0.005926	0.002195	0.002100	0.000361
Guangdong	−0.000842	0.002137	−0.001038	0.002476	0.000640	0.001513	0.000527	0.001799	0.001524	0.001752	−0.000204
Guangxi	−0.009355	0.005425	−0.000342	−0.001270	0.001092	0.001076	0.002802	−0.000304	0.001280	0.001714	−0.000300
Hainan	−0.007454	0.000029	−0.009655	−0.009444	−0.008473	−0.007034	−0.008790	0.002794	−0.006228	−0.005478	−0.009443
Chongqing	0.010658	−0.002033	0.002373	−0.000397	0.000233	0.002647	0.007342	0.021538	0.002083	0.004000	−0.000045
Sichuan	−0.002749	0.001979	−0.002787	−0.001945	−0.001796	0.000007	−0.000878	0.013451	0.001108	0.003624	−0.003425
Guizhou	−0.001220	0.001875	0.000462	0.001401	0.001021	0.000807	0.000445	−0.003049	0.001143	0.001442	0.000605
Yunnan	−0.014332	−0.009768	−0.010508	−0.010440	−0.010063	−0.009341	−0.007497	−0.000388	−0.008753	−0.007916	−0.010445
Shaanxi	−0.002168	−0.000935	0.000113	−0.001778	0.000397	0.002751	0.005545	0.001810	0.003655	0.007311	−0.001383
Gansu	0.000051	−0.001946	0.000551	0.000655	−0.000054	0.001987	0.002605	0.059090	0.002654	0.005858	0.000569
Qinghai	−0.033183	−0.028336	−0.035169	−0.034304	−0.034650	−0.033041	−0.029750	−0.018180	−0.032793	−0.031032	−0.034561
Ningxia	−0.005458	−0.009181	−0.006332	−0.009642	−0.005175	−0.005154	−0.003519	0.001524	−0.004093	−0.003344	−0.006417
Xinjiang	−0.008918	−0.010938	−0.008448	−0.007401	−0.008492	−0.006859	−0.005167	−0.002456	−0.005916	−0.005223	−0.007028

## Supplementary Materials

The following are available online at https://www.mdpi.com/article/10.3390/ijerph182312842/s1, Table S1: Sample learning and prediction accuracy comparison (temperature forecasting VTH), Table S2: Sample learning and prediction accuracy comparison (temperature forecasting OWT (100 million), Table S3: Sample learning and prediction accuracy comparison (temperature forecasting NOH (10,000 people), Table S4: Sample learning and prediction accuracy comparison (humidity forecasting VTH), Table S5: Sample learning and prediction accuracy comparison (humidity forecasting OWT (100 million), Table S6: Sample learning and prediction accuracy comparison (humidity forecasting NOH (10,000 people)), Table S7: Sample learning and prediction accuracy comparison (precipitation forecasting VTH), Table S8: Sample learning and prediction accuracy comparison (precipitation forecasting OWT (100 million), Table S9: Sample learning and prediction accuracy comparison (precipitation forecasting NOH (10,000 people)), Table S10: Sample learning and prediction accuracy comparison (sunshine forecasting VTH), Table S11: Sample learning and prediction accuracy comparison (sunshine forecasting OWT (100 million), Table S12: Sample learning and prediction accuracy comparison (sunshine forecasting NOH (10,000 people)), Table S13: Sample learning and prediction accuracy comparison (four indicators forecasting VTH), Table S14: Sample learning and prediction accuracy comparison (four indicators forecasting OWT (100 million)), Table S15: Sample learning and prediction accuracy comparison (four indicators forecasting NOH: 10,000 people).
