# Supplementary material for: Quantitative Impact Analysis of Climate Change on Residents’ Health Conditions with Improving Eco-Efficiency in China: A Machine Learning Perspective
Source: Int J Environ Res Public Health. 2021 Dec 6;18(23):12842. doi: 10.3390/ijerph182312842 (PMC8657552; doi:10.3390/ijerph182312842)
Supplement: Supplementary file 1 [file ijerph-18-12842-s001.zip › ijerph-1445391-supplementary.pdf]

Table S1. Sample learning and prediction accuracy comparison (temperature forecasting VTH).

Region	MPE	MSE	SDE
**China**	0.000296	0.005300	0.072800
**Beijing**	0.001737	0.000931	0.030512
**Tianjin**	0.001076	0.000051	0.007160
**Hebei**	0.002358	0.000541	0.023249
**Shanxi**	0.000000	0.000000	0.000000
**Inner Mongolia**	0.001108	0.000032	0.005623
**Liaoning**	0.000285	0.000034	0.005794
**Jilin**	0.002201	0.000115	0.010707
**Heilongjiang**	0.001359	0.000125	0.011194
**Shanghai**	0.000032	0.000028	0.005294
**Jiangsu**	0.000000	0.000000	0.000000
**Zhejiang**	0.000002	0.000000	0.000186
**Anhui**	0.000325	0.000008	0.002863
**Fujian**	0.000429	0.000065	0.008038
**Jiangxi**	0.000993	0.000099	0.009925
**Shandong**	0.000094	0.000052	0.007185
**Henan**	0.000160	0.000061	0.007812
**Hubei**	0.000694	0.000102	0.010094
**Hunan**	0.001348	0.000102	0.010115
**Guangdong**	0.000450	0.001427	0.037775
**Guangxi**	0.000156	0.000017	0.004063
**Hainan**	0.000005	0.000000	0.000076
**Chongqing**	0.000000	0.000000	0.000001
**Sichuan**	0.000236	0.000076	0.008695
**Guizhou**	0.000000	0.000000	0.000001
**Yunnan**	0.000050	0.000006	0.002492
**Shaanxi**	0.000001	0.000000	0.000019
**Gansu**	0.002249	0.000068	0.008275
**Qinghai**	0.000019	0.000000	0.000153
**Ningxia**	0.001355	0.000008	0.002798
**Xinjiang**	0.000086	0.000003	0.001823

Table S2. Sample learning and prediction accuracy comparison (temperature forecasting OWT (100 million).

Region	MPE	MSE	SDE
**China**	0.000252	0.004488	0.066990
**Beijing**	0.000871	0.000700	0.026458
**Tianjin**	0.001308	0.000058	0.007612
**Hebei**	0.002730	0.000529	0.023005
**Shanxi**	0.000257	0.000011	0.003264
**Inner Mongolia**	0.001178	0.000029	0.005367
**Liaoning**	0.000273	0.000029	0.005359
**Jilin**	0.001976	0.000091	0.009552
**Heilongjiang**	0.001286	0.000104	0.010191
**Shanghai**	0.000030	0.000025	0.005010
**Jiangsu**	0.000000	0.000000	0.000001
**Zhejiang**	0.000000	0.000000	0.000011
**Anhui**	0.000049	0.000001	0.001220
**Fujian**	0.000583	0.000070	0.008377
**Jiangxi**	0.000844	0.000084	0.009157
**Shandong**	0.000061	0.000029	0.005419
**Henan**	0.000168	0.000056	0.007499
**Hubei**	0.000672	0.000079	0.008904
**Hunan**	0.001355	0.000100	0.009996
**Guangdong**	0.000488	0.001527	0.039079
**Guangxi**	0.000115	0.000012	0.003405
**Hainan**	0.000016	0.000000	0.000349
**Chongqing**	0.000000	0.000000	0.000001
**Sichuan**	0.000272	0.000079	0.008861
**Guizhou**	0.000000	0.000000	0.000000
**Yunnan**	0.000055	0.000008	0.002748
**Shaanxi**	0.000020	0.000000	0.000447
**Gansu**	0.002206	0.000066	0.008107
**Qinghai**	0.001668	0.000004	0.001889
**Ningxia**	0.001226	0.000006	0.002505
**Xinjiang**	0.000079	0.000003	0.001594

Table S3. Sample learning and prediction accuracy comparison (temperature forecasting NOH (10,000 people).

Region	MPE	MSE	SDE
**China**	0.000044	1970.296095	44.388017
**Beijing**	0.000957	19.130206	4.373809
**Tianjin**	0.000119	1.566336	1.251534
**Hebei**	0.002883	254.402122	15.949988
**Shanxi**	0.000260	26.438153	5.141804
**Inner Mongolia**	0.000428	8.783828	2.963752
**Liaoning**	0.000379	16.213131	4.026553
**Jilin**	0.001271	18.086354	4.252805
**Heilongjiang**	0.002088	67.237358	8.199839
**Shanghai**	0.000006	0.047217	0.217294
**Jiangsu**	0.000618	22.453488	4.738511
**Zhejiang**	0.000011	0.024293	0.155861

**Anhui**	0.000267	22.754719	4.770191
**Fujian**	0.001282	35.982166	5.998514
**Jiangxi**	0.000038	0.055206	0.234960
**Shandong**	0.000183	275.718338	16.604769
**Henan**	0.000969	116.294052	10.783972
**Hubei**	0.000223	30.227417	5.497947
**Hunan**	0.001860	86.571669	9.304390
**Guangdong**	0.000386	110.342589	10.504408
**Guangxi**	0.000001	0.002497	0.049968
**Hainan**	0.000211	0.437663	0.661561
**Chongqing**	0.000341	8.059000	2.838838
**Sichuan**	0.000444	135.583188	11.644019
**Guizhou**	0.000000	0.000000	0.000018
**Yunnan**	-0.000068	46.753858	6.837679
**Shaanxi**	0.000101	2.743315	1.656296
**Gansu**	0.002920	27.769131	5.269642
**Qinghai**	0.000064	0.000424	0.020597
**Ningxia**	0.001123	2.183168	1.477555
**Xinjiang**	0.000956	26.025765	5.101545

Table S4. Sample learning and prediction accuracy comparison (humidity forecasting VTH).

Region	MPE	MSE	SDE
**China**	0.000259	0.004053	0.063664
**Beijing**	0.001203	0.000275	0.016584
**Tianjin**	0.000333	0.000020	0.004513
**Hebei**	0.000389	0.000059	0.007697
**Shanxi**	0.000125	0.000004	0.002043
**Inner Mongolia**	0.000333	0.000006	0.002459
**Liaoning**	0.000404	0.000078	0.008833
**Jilin**	0.001250	0.000069	0.008297
**Heilongjiang**	0.001241	0.000123	0.011094
**Shanghai**	0.000035	0.000015	0.003911
**Jiangsu**	0.000000	0.000000	0.000013
**Zhejiang**	0.000083	0.000066	0.008116
**Anhui**	0.000065	0.000002	0.001411
**Fujian**	0.000354	0.000041	0.006384
**Jiangxi**	0.000590	0.000017	0.004141
**Shandong**	0.000565	0.000645	0.025397
**Henan**	0.000172	0.000041	0.006368
**Hubei**	0.000580	0.000085	0.009219
**Hunan**	0.000000	0.000000	0.000000
**Guangdong**	0.000127	0.000335	0.018299
**Guangxi**	0.000838	0.000117	0.010802
**Hainan**	0.000314	0.000001	0.001121
**Chongqing**	0.000973	0.000025	0.004960
**Sichuan**	0.000521	0.000173	0.013141
**Guizhou**	0.000032	0.000000	0.000472
**Yunnan**	0.000000	0.000000	0.000314
**Shaanxi**	0.001042	0.000080	0.008928

**Gansu**	0.002010	0.000081	0.009019
**Qinghai**	0.000019	0.000000	0.000083
**Ningxia**	0.001141	0.000007	0.002628
**Xinjiang**	0.000353	0.000018	0.004284

Table S5. Sample learning and prediction accuracy comparison (humidity forecasting OWT (100 million).

Region	MPE	MSE	SDE
**China**	0.003374	0.058088	0.003374
**Beijing**	0.000257	0.016031	0.000257
**Tianjin**	0.000001	0.001164	0.000001
**Hebei**	0.000019	0.004318	0.000019
**Shanxi**	0.000008	0.002830	0.000008
**Inner Mongolia**	0.000002	0.001506	0.000002
**Liaoning**	0.000077	0.008758	0.000077
**Jilin**	0.000069	0.008280	0.000069
**Heilongjiang**	0.000098	0.009903	0.000098
**Shanghai**	0.000014	0.003755	0.000014
**Jiangsu**	0.000000	0.000022	0.000000
**Zhejiang**	0.000076	0.008734	0.000076
**Anhui**	0.000000	0.000136	0.000000
**Fujian**	0.000037	0.006053	0.000037
**Jiangxi**	0.000014	0.003799	0.000014
**Shandong**	0.000565	0.023771	0.000565
**Henan**	0.000039	0.006274	0.000039
**Hubei**	0.000067	0.008160	0.000067
**Hunan**	0.000000	0.000000	0.000000
**Guangdong**	0.001636	0.040452	0.001636
**Guangxi**	0.000044	0.006657	0.000044
**Hainan**	0.000001	0.000777	0.000001
**Chongqing**	0.000053	0.007266	0.000053
**Sichuan**	0.000141	0.011886	0.000141
**Guizhou**	0.000000	0.000000	0.000000
**Yunnan**	0.000000	0.000647	0.000000
**Shaanxi**	0.000078	0.008829	0.000078
**Gansu**	0.000076	0.008704	0.000076
**Qinghai**	0.000005	0.002133	0.000005
**Ningxia**	0.000005	0.002324	0.000005
**Xinjiang**	0.000033	0.005712	0.000033

Table S6. Sample learning and prediction accuracy comparison (humidity forecasting NOH (10,000 people)).

Region	MPE	MSE	SDE
**China**	0.000135	1630.011984	40.373407
**Beijing**	0.001182	22.630093	4.757110
**Tianjin**	0.000430	2.786358	1.669239
**Hebei**	0.004929	421.082367	20.520292
**Shanxi**	0.000176	29.496203	5.431041
**Inner Mongolia**	0.000150	7.473908	2.733845
**Liaoning**	0.000294	21.336610	4.619157
**Jilin**	0.000965	13.713701	3.703201
**Heilongjiang**	0.001794	78.336723	8.850804
**Shanghai**	0.000049	1.686020	1.298468
**Jiangsu**	0.000989	152.752013	12.359289
**Zhejiang**	0.000019	0.486412	0.697432
**Anhui**	0.000000	0.000001	0.000801
**Fujian**	0.001600	26.933498	5.189749
**Jiangxi**	0.000863	73.052363	8.547067
**Shandong**	0.000567	354.972466	18.840713
**Henan**	0.000835	80.442702	8.968986
**Hubei**	0.000735	39.259898	6.265772
**Hunan**	0.001931	85.982476	9.272674
**Guangdong**	0.001496	137.847088	11.740830
**Guangxi**	0.000005	0.361892	0.601575
**Hainan**	0.000066	0.052808	0.229800
**Chongqing**	0.000469	1.807055	1.344268
**Sichuan**	0.000405	126.769778	11.259209
**Guizhou**	0.000453	7.685597	2.772291
**Yunnan**	-0.000196	52.116854	7.219200
**Shaanxi**	0.000000	0.000000	0.000498
**Gansu**	0.002448	30.362087	5.510180
**Qinghai**	0.012486	6.584755	2.566078
**Ningxia**	0.001381	1.278189	1.130570
**Xinjiang**	0.001377	32.254166	5.679275

Table S7. Sample learning and prediction accuracy comparison (precipitation forecasting VTH).

Region	MPE	MSE	SDE
**China**	0.000296	0.004604	0.067855
**Beijing**	0.002875	0.000797	0.028236
**Tianjin**	0.000378	0.000028	0.005317
**Hebei**	0.001867	0.000328	0.018121
**Shanxi**	0.001259	0.000075	0.008634
**Inner Mongolia**	0.000247	0.000003	0.001646
**Liaoning**	0.000258	0.000046	0.006770
**Jilin**	0.001905	0.000122	0.011028
**Heilongjiang**	0.001349	0.000126	0.011217
**Shanghai**	0.000063	0.000036	0.005987
**Jiangsu**	0.000016	0.000010	0.003100
**Zhejiang**	0.000175	0.000114	0.010688

**Anhui**	0.000888	0.000049	0.006969
**Fujian**	0.000420	0.000073	0.008520
**Jiangxi**	0.000598	0.000021	0.004585
**Shandong**	0.000434	0.000432	0.020785
**Henan**	0.000410	0.000082	0.009052
**Hubei**	0.001194	0.000172	0.013099
**Hunan**	0.000083	0.000006	0.002456
**Guangdong**	0.000305	0.001057	0.032512
**Guangxi**	0.000570	0.000087	0.009348
**Hainan**	0.000000	0.000000	0.000001
**Chongqing**	0.000000	0.000000	0.000014
**Sichuan**	0.000237	0.000061	0.007808
**Guizhou**	0.001172	0.000040	0.006293
**Yunnan**	0.000062	0.000010	0.003205
**Shaanxi**	0.001060	0.000080	0.008949
**Gansu**	0.000001	0.000000	0.000008
**Qinghai**	0.000019	0.000000	0.000242
**Ningxia**	0.001506	0.000008	0.002902
**Xinjiang**	0.000122	0.000010	0.003126

Table S8. Sample learning and prediction accuracy comparison (precipitation forecasting OWT (100 million).

Region	MPE	MSE	SDE
**China**	0.000252	0.003812	0.061742
**Beijing**	0.002094	0.000603	0.024547
**Tianjin**	0.000429	0.000031	0.005571
**Hebei**	0.001320	0.000146	0.012099
**Shanxi**	0.000835	0.000043	0.006571
**Inner Mongolia**	0.000015	0.000000	0.000159
**Liaoning**	0.000262	0.000077	0.008776
**Jilin**	0.001682	0.000102	0.010076
**Heilongjiang**	0.001343	0.000115	0.010725
**Shanghai**	0.000056	0.000033	0.005729
**Jiangsu**	0.000004	0.000003	0.001628
**Zhejiang**	0.000138	0.000080	0.008918
**Anhui**	0.000101	0.000006	0.002364
**Fujian**	0.000507	0.000061	0.007795
**Jiangxi**	0.000528	0.000017	0.004169
**Shandong**	0.000312	0.000260	0.016126
**Henan**	0.000316	0.000062	0.007893
**Hubei**	0.001011	0.000124	0.011134
**Hunan**	0.000113	0.000008	0.002827
**Guangdong**	0.000313	0.001010	0.031788
**Guangxi**	0.000306	0.000041	0.006410
**Hainan**	0.000069	0.000000	0.000528
**Chongqing**	0.000000	0.000000	0.000016
**Sichuan**	0.000251	0.000066	0.008122
**Guizhou**	0.000156	0.000002	0.001230
**Yunnan**	0.000040	0.000007	0.002589
**Shaanxi**	0.001244	0.000075	0.008649

**Gansu**	0.000000	0.000000	0.000000
**Qinghai**	0.000002	0.000000	0.000008
**Ningxia**	0.001396	0.000007	0.002643
**Xinjiang**	0.000149	0.000008	0.002911

Table S9. Sample learning and prediction accuracy comparison (precipitation forecasting NOH (10,000 people)).

Region	MPE	MSE	SDE
**China**	0.000121	1584.344982	39.803831
**Beijing**	0.001689	19.076299	4.367642
**Tianjin**	0.000245	1.713238	1.308907
**Hebei**	0.004611	370.160030	19.239543
**Shanxi**	0.000355	23.198834	4.816517
**Inner Mongolia**	0.000227	4.596486	2.143942
**Liaoning**	0.000396	34.112284	5.840572
**Jilin**	0.001430	21.209976	4.605429
**Heilongjiang**	0.002159	81.322054	9.017874
**Shanghai**	0.000046	2.030433	1.424933
**Jiangsu**	0.000426	7.774070	2.788202
**Zhejiang**	0.000000	0.000000	0.000515
**Anhui**	0.000194	9.486102	3.079952
**Fujian**	0.001784	69.433223	8.332660
**Jiangxi**	0.001406	11.782794	3.432607
**Shandong**	0.000274	316.463392	17.789418
**Henan**	0.000963	111.124734	10.541572
**Hubei**	0.000932	43.724231	6.612430
**Hunan**	0.000548	18.438753	4.294037
**Guangdong**	0.000449	120.761869	10.989171
**Guangxi**	-0.000071	20.839091	4.564985
**Hainan**	0.000054	0.046119	0.214754
**Chongqing**	-0.000257	8.732400	2.955063
**Sichuan**	0.000000	0.000000	0.000233
**Guizhou**	0.000267	2.703736	1.644304
**Yunnan**	0.000704	60.457944	7.775471
**Shaanxi**	0.000056	2.661778	1.631496
**Gansu**	0.000000	0.000000	0.000130
**Qinghai**	0.014937	5.599743	2.366378
**Ningxia**	0.002009	2.335538	1.528247
**Xinjiang**	0.001669	39.287940	6.268009

Table S10. Sample learning and prediction accuracy comparison (sunshine forecasting VTH).

Region	MPE	MSE	SDE
**China**	0.000165	0.001513	0.038903
**Beijing**	0.000882	0.000330	0.018173
**Tianjin**	0.000074	0.000003	0.001620
**Hebei**	0.001361	0.000250	0.015825
**Shanxi**	0.000000	0.000000	0.000000
**Inner Mongolia**	0.000327	0.000007	0.002645
**Liaoning**	0.000374	0.000053	0.007292

**Jilin**	0.000000	0.000000	0.000000
**Heilongjiang**	0.001443	0.000120	0.010948
**Shanghai**	0.000068	0.000054	0.007322
**Jiangsu**	0.000083	0.000093	0.009654
**Zhejiang**	0.000208	0.000087	0.009320
**Anhui**	0.000127	0.000009	0.003052
**Fujian**	0.000553	0.000049	0.007027
**Jiangxi**	0.000325	0.000012	0.003467
**Shandong**	0.000040	0.000006	0.002366
**Henan**	0.000154	0.000049	0.007002
**Hubei**	0.000408	0.000042	0.006493
**Hunan**	0.001044	0.000068	0.008268
**Guangdong**	0.000454	0.001836	0.042854
**Guangxi**	0.000438	0.000063	0.007935
**Hainan**	0.000000	0.000000	0.000003
**Chongqing**	0.000000	0.000000	0.000006
**Sichuan**	0.000247	0.000109	0.010424
**Guizhou**	0.002262	0.000067	0.008161
**Yunnan**	0.000029	0.000003	0.001842
**Shaanxi**	0.000000	0.000000	0.000000
**Gansu**	0.001400	0.000045	0.006739
**Qinghai**	0.000091	0.000000	0.000626
**Ningxia**	0.001394	0.000007	0.002728
**Xinjiang**	0.000349	0.000022	0.004742

Table S11. Sample learning and prediction accuracy comparison (sunshine forecasting OWT (100 million).

Region	MPE	MSE	SDE
**China**	0.000146	0.001499	0.038719
**Beijing**	0.000593	0.000310	0.017600
**Tianjin**	0.000193	0.000012	0.003432
**Hebei**	0.000088	0.000004	0.001979
**Shanxi**	0.000678	0.000042	0.006481
**Inner Mongolia**	0.000451	0.000008	0.002835
**Liaoning**	0.000040	0.000001	0.001182
**Jilin**	0.002646	0.000118	0.010863
**Heilongjiang**	0.000909	0.000061	0.007821
**Shanghai**	0.000050	0.000053	0.007314
**Jiangsu**	0.000050	0.000048	0.006942
**Zhejiang**	0.000078	0.000032	0.005692
**Anhui**	0.000157	0.000046	0.006777
**Fujian**	0.000522	0.000038	0.006196
**Jiangxi**	0.000419	0.000017	0.004170
**Shandong**	0.000017	0.000001	0.001011
**Henan**	0.000151	0.000042	0.006507
**Hubei**	0.000342	0.000027	0.005175
**Hunan**	0.001053	0.000064	0.008024
**Guangdong**	0.000446	0.001672	0.040890
**Guangxi**	0.000250	0.000031	0.005544
**Hainan**	0.000171	0.000001	0.000925

**Chongqing**	0.000218	0.000030	0.005504
**Sichuan**	0.000277	0.000115	0.010725
**Guizhou**	0.000411	0.000007	0.002733
**Yunnan**	0.000051	0.000005	0.002182
**Shaanxi**	0.000026	0.000000	0.000634
**Gansu**	0.001853	0.000052	0.007244
**Qinghai**	0.002072	0.000004	0.002106
**Ningxia**	0.001103	0.000006	0.002353
**Xinjiang**	0.000446	0.000025	0.004955

Table S12. Sample learning and prediction accuracy comparison (sunshine forecasting NOH (10,000 people)).

Region	MPE	MSE	SDE
**China**	0.000043	270.196617	16.437658
**Beijing**	0.001227	18.586989	4.311263
**Tianjin**	0.000272	2.179508	1.476316
**Hebei**	0.004527	335.369559	18.313098
**Shanxi**	0.000141	35.946415	5.995533
**Inner Mongolia**	0.000588	11.474070	3.387340
**Liaoning**	0.000118	13.013918	3.607481
**Jilin**	0.001620	17.995828	4.242149
**Heilongjiang**	0.000877	24.101399	4.909318
**Shanghai**	0.000054	2.678408	1.636584
**Jiangsu**	0.000455	45.777794	6.765929
**Zhejiang**	0.000000	0.000000	0.000044
**Anhui**	-0.000231	35.627764	5.968900
**Fujian**	0.003298	40.040784	6.327779
**Jiangxi**	0.000087	1.203376	1.096985
**Shandong**	0.000533	295.974136	17.203899
**Henan**	0.000335	35.615976	5.967912
**Hubei**	0.001495	65.521461	8.094533
**Hunan**	0.002869	101.089104	10.054308
**Guangdong**	0.002227	224.810225	14.993673
**Guangxi**	0.000033	11.781165	3.432370
**Hainan**	0.000081	0.598349	0.773530
**Chongqing**	0.000000	0.000001	0.000784
**Sichuan**	0.000229	182.066516	13.493203
**Guizhou**	0.000874	21.418014	4.627960
**Yunnan**	0.000191	17.352971	4.165690
**Shaanxi**	0.000026	2.512223	1.584999
**Gansu**	0.002432	28.259861	5.316001
**Qinghai**	0.017046	7.760332	2.785737
**Ningxia**	0.001664	2.053189	1.432895
**Xinjiang**	0.000907	18.915541	4.349200

Table S13. Sample learning and prediction accuracy comparison (four indicators forecasting VTH).

Region	MPE	MSE	SDE
**China**	0.000811	0.010810	0.103971
**Beijing**	0.001013	0.000203	0.014242

**Tianjin**	0.000000	0.000000	0.000000
**Hebei**	0.001254	0.000258	0.016048
**Shanxi**	0.000000	0.000000	0.000002
**Inner Mongolia**	0.000003	0.000000	0.000031
**Liaoning**	0.000623	0.000056	0.007515
**Jilin**	0.000000	0.000000	0.000000
**Heilongjiang**	0.000485	0.000020	0.004454
**Shanghai**	0.000000	0.000000	0.000036
**Jiangsu**	0.000639	0.000819	0.028615
**Zhejiang**	0.004995	0.003639	0.060321
**Anhui**	0.000000	0.000000	0.000000
**Fujian**	0.000000	0.000000	0.000017
**Jiangxi**	0.000563	0.000055	0.007400
**Shandong**	0.000310	0.000332	0.018208
**Henan**	0.000000	0.000000	0.000000
**Hubei**	0.000000	0.000000	0.000000
**Hunan**	0.000672	0.000058	0.007636
**Guangdong**	0.000290	0.000888	0.029797
**Guangxi**	0.000443	0.000037	0.006108
**Hainan**	0.000000	0.000000	0.000000
**Chongqing**	0.000970	0.000028	0.005302
**Sichuan**	0.000379	0.000160	0.012652
**Guizhou**	0.000000	0.000000	0.000000
**Yunnan**	0.000001	0.000000	0.000132
**Shaanxi**	0.000706	0.000044	0.006650
**Gansu**	0.001845	0.000058	0.007648
**Qinghai**	0.000000	0.000000	0.000000
**Ningxia**	0.001917	0.000007	0.002560
**Xinjiang**	0.000226	0.000011	0.003317

Table S14. Sample learning and prediction accuracy comparison (four indicators forecasting OWT (100 million)).

Region	MPE	MSE	SDE
**China**	0.000651	0.010340	0.101683
**Beijing**	0.001128	0.000240	0.015485
**Tianjin**	0.000000	0.000000	0.000000
**Hebei**	0.001368	0.000220	0.014841
**Shanxi**	0.000000	0.000000	0.000004
**Inner Mongolia**	0.000001	0.000000	0.000011
**Liaoning**	0.000342	0.000018	0.004298
**Jilin**	0.001028	0.000049	0.007010
**Heilongjiang**	0.000213	0.000005	0.002254
**Shanghai**	0.000000	0.000000	0.000003
**Jiangsu**	0.000022	0.000019	0.004314
**Zhejiang**	0.005363	0.003790	0.061564
**Anhui**	0.000207	0.000008	0.002796
**Fujian**	0.000000	0.000000	0.000008
**Jiangxi**	0.000000	0.000000	0.000000
**Shandong**	0.000242	0.000296	0.017211
**Henan**	0.000000	0.000000	0.000000

**Hubei**	0.000000	0.000000	0.000000
**Hunan**	0.000563	0.000057	0.007576
**Guangdong**	0.000280	0.000776	0.027856
**Guangxi**	0.000295	0.000018	0.004296
**Hainan**	0.000197	0.000001	0.001000
**Chongqing**	0.001036	0.000028	0.005333
**Sichuan**	0.000414	0.000149	0.012222
**Guizhou**	0.000000	0.000000	0.000000
**Yunnan**	0.000000	0.000000	0.000015
**Shaanxi**	0.000748	0.000037	0.006109
**Gansu**	0.001971	0.000053	0.007294
**Qinghai**	0.002579	0.000004	0.001918
**Ningxia**	0.001461	0.000008	0.002795
**Xinjiang**	0.000110	0.000004	0.001999

Table S15. Sample learning and prediction accuracy comparison (four indicators forecasting NOH: 10,000 people).

Region	MPE	MSE	SDE
**China**	0.000002	0.066784	0.258426
**Beijing**	0.000644	10.606083	3.256698
**Tianjin**	0.004849	4.744277	2.178136
**Hebei**	0.001584	190.051158	13.785904
**Shanxi**	0.000215	9.629034	3.103069
**Inner Mongolia**	0.000001	0.001251	0.035367
**Liaoning**	0.000000	0.000000	0.000585
**Jilin**	0.001054	9.280906	3.046458
**Heilongjiang**	0.000748	17.888213	4.229446
**Shanghai**	0.000118	3.459122	1.859871
**Jiangsu**	0.000402	21.905976	4.680382
**Zhejiang**	0.000128	8.231969	2.869141
**Anhui**	0.000517	29.993593	5.476641
**Fujian**	0.002244	79.058099	8.891462
**Jiangxi**	0.000000	0.000000	0.000111
**Shandong**	0.000287	205.890915	14.348899
**Henan**	0.000000	0.000030	0.005500
**Hubei**	0.008690	255.397376	15.981157
**Hunan**	0.001632	59.442125	7.709872
**Guangdong**	0.001153	80.864327	8.992459
**Guangxi**	0.000312	0.968018	0.983879
**Hainan**	0.003177	1.927869	1.388477
**Chongqing**	0.000000	0.000000	0.000210
**Sichuan**	0.000348	173.464960	13.170610
**Guizhou**	0.000000	0.000000	0.000264
**Yunnan**	0.000694	44.667220	6.683354
**Shaanxi**	0.000000	0.000452	0.021260
**Gansu**	0.002734	24.762121	4.976155
**Qinghai**	0.012505	6.635588	2.575964
**Ningxia**	0.002133	1.081430	1.039918
**Xinjiang**	0.003732	46.643747	6.829623
